# A Cilia–Centered Nicotinamide Adenine Dinucleotide (NAD ^+^) Signaling Network Regulates Hypothalamic Lysosomal Homeostasis and Metabolic Aging

**DOI:** 10.1111/acel.70745

**Published:** 2026-10-20

**Authors:** Se Eun Park, Eun Roh, Jae Woo Park, Seong Jun Kim, So Yeon Jeong, Chae Beom Park, Hyo Sun Lim, Ji Soo Kim, Mun Gyeom Kim, Myeong Ju Kim, Young In Cho, Wontae Jeong, Gil Myoung Kang, Sang‐Yeob Kim, Se Hee Min, Min‐Seon Kim

**Affiliations:** ^1^ Department of Biomedical Science University of Ulsan College of Medicine Seoul Korea; ^2^ Division of Endocrinology and Metabolism, Department of Internal Medicine, Boramae Medical Center Seoul National University College of Medicine Seoul Korea; ^3^ Asan Institute for Life Science, Asan Medical Center Seoul Korea; ^4^ Division of Endocrinology and Metabolism, Diabetes Center, Asan Medical Center University of Ulsan College of Medicine Seoul Korea

**Keywords:** aging, calorie restriction, CHMP4B, hypothalamic neurons, lysosomal homeostasis, NAD^+^, primary cilia, TRPML1

## Abstract

Nicotinamide adenine dinucleotide (NAD^+^) is an essential metabolite that regulates diverse cellular metabolic processes. Primary cilia, hair–like organelles on the cell surface, function as critical signaling hubs. Here, we demonstrate that cellular NAD^+^ levels are key regulators of both primary cilia growth and lysosomal function in hypothalamic neurons. NAD^+^ depletion induces ciliary shortening and diminishes lysosomal activity in a cilia–dependent manner. Mechanistically, the lysosomal repair complex protein CHMP4B translocates from periciliary satellites to lysosomes during lysosomal stress, a process impaired by ciliary dysgenesis. Disrupted lysosomal recruitment of CHMP4B leads to abnormal lysosomal Ca^2+^ signaling and lysosomal dysfunction. Aged mice with hypothalamic NAD^+^ deficiency exhibit shortened cilia and lysosomal stress phenotypes, both of which are restored by calorie restriction (CR). Moreover, CR improves aging–associated metabolic dysfunction, at least in part, through hypothalamic cilia–dependent mechanisms. These findings identify a primary cilium–mediated–NAD^+^ signaling axis that maintains lysosomal homeostasis in hypothalamic neurons, the disruption of which drives metabolic aging.

## Graphical abstract

Aging‐associated NAD^+^ depletion in hypothalamic neurons shortens primary cilia and disrupts CHMP4B trafficking to lysosomes during lysosomal stress, leading to impaired TRPML1–mediated Ca^2+^ signaling and lysosomal stress responses. Calorie restriction restores hypothalamic NAD^+^ levels, ciliary structure, and lysosomal homeostasis, thereby improving aging–associated metabolic dysfunction through a hypothalamic cilia–dependent mechanism.

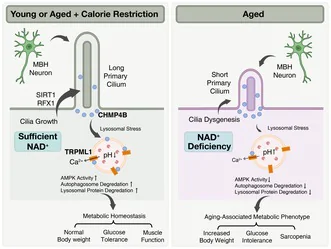

## 1 Introduction

Nicotinamide adenine dinucleotide (NAD^+^) plays a pivotal role in maintaining cellular redox homeostasis (Belenky et al. 2007) and supports numerous biochemical processes mediated by NAD^+^–dependent enzymes, including sirtuins (SIRT1–7), poly (ADP–ribose) polymerases (PARPs), and CD38 (Imai and Guarente 2014; Xie et al. 2020). In mammalian cells, intracellular NAD^+^ levels are primarily sustained through the salvage pathway (Xie et al. 2020), wherein NAD^+^ is resynthesized from nicotinamide via the intermediate nicotinamide mononucleotide (NMN). The rate–limiting step of this pathway is catalyzed by nicotinamide phosphoribosyltransferase (NAMPT), which converts nicotinamide into NMN (Garten et al. 2015; Revollo et al. 2007). Therefore, suppression of NAMPT activity markedly reduces intracellular NAD^+^ availability (Garten et al. 2015; Revollo et al. 2007).

Aging is a well–established risk factor for systemic metabolic dysfunctions, including obesity, hyperglycemia, insulin resistance, and sarcopenia (Batsis and Villareal 2018). Among the brain regions affected by aging, the hypothalamus has emerged as a critical node that coordinates energy balance, endocrine function, and whole–body homeostasis (Roh et al. 2016; Zhang et al. 2013). As a central integrator of internal metabolic signals and external environmental cues, the hypothalamus plays a key role in orchestrating the systemic aging process (Zhang et al. 2013). Age–related decline in NAD^+^ levels has been linked to impaired mitochondrial function, reduced neuronal activity, and increased susceptibility to metabolic and degenerative diseases (Fang et al. 2017; Imai and Guarente 2014; Verdin 2015). In the hypothalamus, the NAD^+^–dependent deacetylase SIRT1 has been shown to promote longevity by activating neurons in the lateral and dorsomedial hypothalamic regions (Satoh et al. 2013), underscoring the functional importance of NAD^+^ homeostasis in this part of the brain.

Most mammalian cells, including hypothalamic neurons, have microtubule–based antenna–like organelles called primary cilia on their cell surfaces (Singla and Reiter 2006). These primary cilia serve as sensors of the extracellular environment and are essential for transducing signals and maintaining cellular homeostasis (Singla and Reiter 2006). Ciliary dysfunction is a hallmark of several genetic disorders characterized by obesity and insulin resistance, including Bardet–Biedl and Alström syndromes (Lee et al. 2022), which are now classified as genetic ciliopathies. Moreover, experimental inhibition of ciliary growth in hypothalamic neurons leads to hyperphagia and impaired energy balance, supporting a role for primary cilia in hypothalamic metabolic regulation (Davenport et al. 2007). Notably, a recent study demonstrated that cilia bearing the melanocortin 4 receptor (MC4R) in hypothalamic paraventricular neurons shorten with age, contributing to obesity and leptin resistance (Oya et al. 2024). However, the biological mechanisms underlying these age–related ciliary changes remain largely unknown.

Lysosomes are essential organelles that play a critical role in maintaining cellular homeostasis by degrading damaged proteins, lipids, and organelles through hydrolytic enzymes (Ballabio and Bonifacino 2020). They receive this cargo via autophagy, which sequesters cellular waste into autophagosomes that subsequently fuse with lysosomes for final breakdown (Ballabio and Bonifacino 2020). Neurons, being post–mitotic and long‐lived, are particularly dependent on efficient autophagic flux and lysosomal function to prevent the accumulation of toxic substrates (Rubinsztein et al. 2005). Since disruption of lysosomal functional integrity may contribute to age–related neurodegeneration, enhancing lysosomal function has emerged as a promising therapeutic strategy (Udayar et al. 2022). Interestingly, a functional and anatomical link between primary cilia and autophagy has been demonstrated, revealing a reciprocal interaction between the two systems (Orhon et al. 2015; Pampliega et al. 2013). A recent study also reported that primary cilia in hippocampal neurons mediate autophagy regulation in response to the bone‐derived hormone osteocalcin via GPR158–cAMP signaling (Rivagorda et al. 2025).

NAD^+^ has been implicated in the regulation of autophagy through SIRT1–mediated deacetylation of autophagy–related proteins (Lee et al. 2008). However, it remains unclear whether NAD^+^ also plays a role in regulating cilia structures and cilia–associated signaling pathways. Given that a decline in cellular NAD^+^ levels is closely associated with various aging–related pathologies (Fang et al. 2017; Imai and Guarente 2014), we investigated the relationship between NAD^+^ biology and primary cilia in hypothalamic neurons. Our findings indicate that NAD^+^ is a potent regulator of ciliary growth. Moreover, our study demonstrates a potential cilia–mediated mechanism by which NAD^+^ decline leads to lysosomal dysfunction in the aging hypothalamus, thereby promoting aging–associated metabolic phenotypes.

## 2 Results

### 2.1 NAD^+^ Depletion Causes Aging–Related Short Cilia Phenotype in the Hypothalamic Neurons

We first investigated whether age–related NAD^+^ decline is associated with alterations in primary cilia morphology in the mediobasal hypothalamus (MBH), which has been proposed as a regulatory center for systemic aging (Zhang et al. 2013). We analyzed ciliary length in MBH sections from young (3–month–old) and aged (24–month–old) mice. Immunostaining for the neuronal ciliary marker adenylyl cyclase–3 (AC3) (Han et al. 2014) revealed that young mice exhibited relatively long primary cilia in the MBH (Figure 1a), with an average length of approximately 7.4 μm. We observed a significant shortening of primary cilia in the MBH of aged mice compared with young controls, without changes in the percentage of ciliated cells (Figure 1a). To determine whether these ciliary changes were associated with altered NAD^+^ availability, we measured hypothalamic NAD^+^ levels in both groups. As expected, aged mice showed a marked reduction in hypothalamic NAD^+^ content relative to young mice (Figure 1b).

**FIGURE 1:**
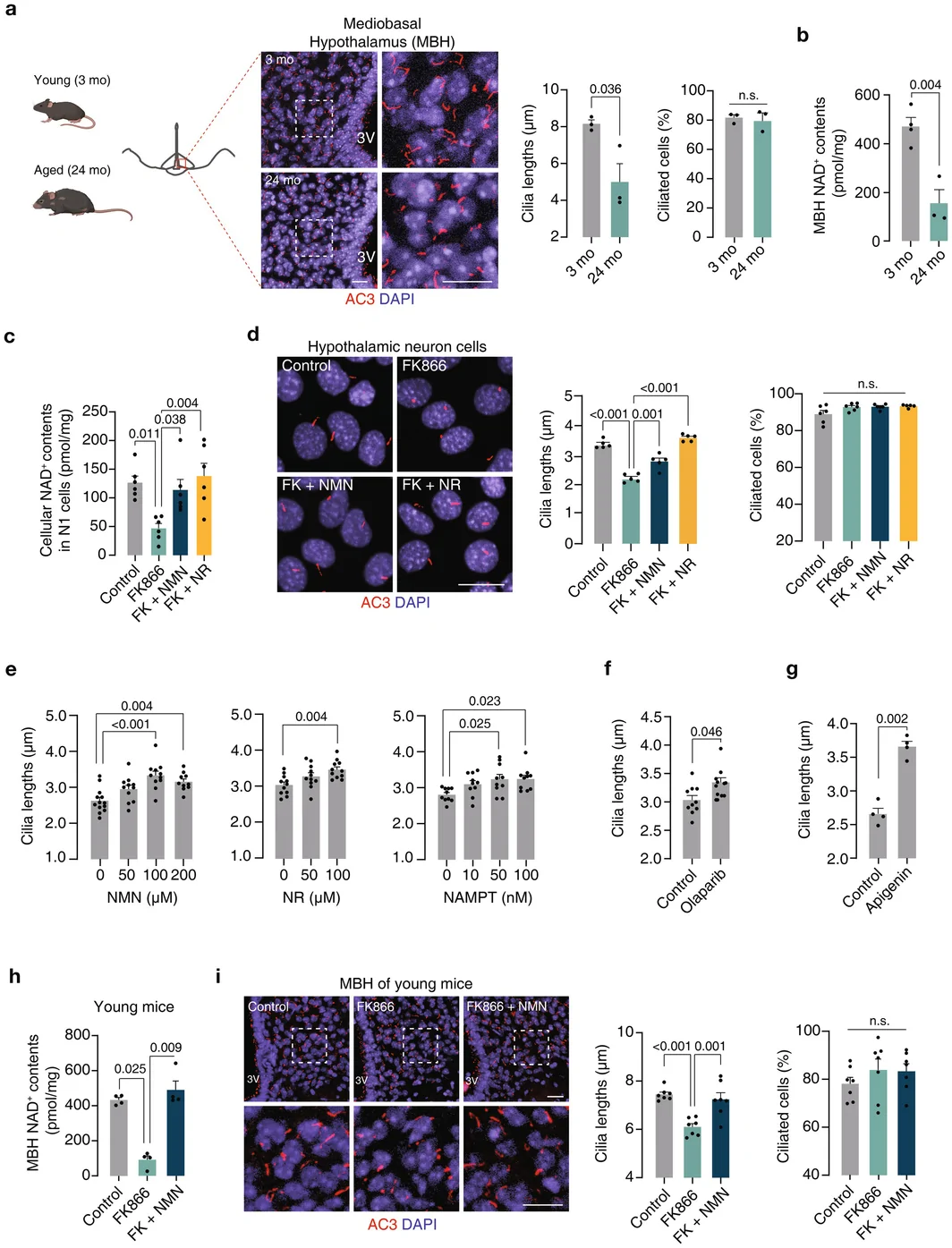
NAD^+^ depletion induces aging‐related ciliary shortening in hypothalamic neurons. (a) Aged mice (24 months old) showed shorter primary cilia in the mediobasal hypothalamus (MBH) compared with young mice (3 months old), with no change in the percentage of ciliated cells (*n* = 3 mice per group). AC3, adenylyl cyclase 3. (b) Hypothalamic NAD^+^ levels were significantly reduced in aged mice compared with young mice (*n* = 3–4 mice per group). (c) Cellular NAD^+^ levels in N1 hypothalamic neuron cells following treatment with FK866 (10 nM), with or without nicotinamide mononucleotide (NMN, 100 μM) or nicotinamide ribose (NR, 100 μM) (*n* = 6 wells per group). (d) FK866 treatment shortened cilia in N1 cells, an effect that was rescued by co–treatment with NMN or NR without altering the percentage of ciliated cells (*n* = 5 wells per group). (e) Effects of treatment with NAD^+^ precursors (MNN or NR) or NAMPT peptide on cilia length in N1 cells (*n* = 10–13 wells per group). (f, g) Treatment with the PARP inhibitor olaparib and the CD38 inhibitor apigenin elongated cilia in hypothalamic neurons (f; *n* = 10 wells, g; *n* = 4 wells per group). (h, i) Effects of 5–day intracerebroventricular administration of FK866, with or without NMN, on hypothalamic NAD^+^ contents (*n* = 4 mice) and MBH cilia (*n* = 3000 cilia from 7 mice per group). Data were obtained from three independent experiments (c–i), except for the aging cohort experiments (a, b), due to the limited availability of aged mice. Data are presented as the mean ± SEM. Statistical analyses were performed using two‐sided unpaired Student's *t*–test (a–right, b, f, g), one–way ANOVA (c, d–right, e, h, i–right), or two–way ANOVA (a–left, d–left, i–left) with Tukey's post hoc test. n.s., not significant. Scale bars, 20 μm.

We next conducted in vitro experiments to determine whether changes in cellular NAD^+^ levels affect primary cilia in N1 hypothalamic neuronal cells. We induced ciliogenesis by culturing cells under confluent and serum–deprived conditions for 24 h. We then depleted cellular NAD^+^ levels by treating the cells with FK866, a chemical inhibitor of NAD^+^ biosynthetic enzyme NAMPT (Hasmann and Schemainda 2003), for 16 h. Successful NAD^+^ depletion in these cells was confirmed (Figure 1c). FK866 treatment led to a significant reduction in primary cilia length, while the proportion of ciliated cells remained unchanged (Figure 1d). These results suggest that NAD^+^ depletion shortens cilia without affecting ciliogenesis in hypothalamic neuron cells. Under these conditions, FK866 treatment did not significantly induce cell death or cellular stress (Figure S1), indicating that FK866–induced ciliary changes were not caused by nonspecific cellular stress. Furthermore, restoring NAD^+^ levels through supplementation with nicotinamide mononucleotide (NMN) and nicotinamide riboside (NR), precursor in the NAD^+^ salvage pathway (Xie et al. 2020), successfully reversed ciliary shortening (Figure 1c,d). In addition, elevating NAD^+^ levels through treatment with NMN or NR, as well as the NAMPT peptide, increased cilia length even in FK866–untreated serum–starved cells, suggesting that boosting NAD^+^ levels, in addition to serum starvation, further stimulates ciliary growth (Figure 1e). Treatment with olaparib, a PARP inhibitor that elevates intracellular NAD^+^ by reducing its PARP–mediated consumption (Xie et al. 2020; Zheng et al. 2020), significantly increased ciliary length in N1 cells (Figure 1f). Similarly, apigenin, a known inhibitor of another NAD^+^ consuiming enzyme CD38 (Escande et al. 2013; Imai and Guarente 2014), also promoted cilia growth (Figure 1g). These findings demonstrate that NAD^+^ is a critical regulator of primary cilium length in hypothalamic neurons. We next examined whether NAD^+^ depletion and repletion similarly affect primary cilia in other cell types. Comparable changes in cilium length in response to altered NAD^+^ levels were observed in retinal pigment epithelial (RPE–1) cells (Figure S2). Together, these findings suggest that NAD^+^ is a conserved regulator of primary cilium length across diverse cell types.

We further examined whether alterations in NAD^+^ levels affect primary cilia length in the MBH of young adult mice. FK866 was administered into the third ventricle of 3–month–old mice to inhibit NAD^+^ biosynthesis. To assess reversibility, a separate group of mice received NMN co–administration together with FK866. Intracerebroventricular FK866 treatment markedly reduced MBH NAD^+^ levels, whereas NMN co–supplementation prevented FK866–induced NAD^+^ depletion (Figure 1h). Consistent with our in vitro findings (Figure 1d), pharmacological depletion and repletion of NAD^+^ significantly altered primary cilia length without affecting the percentage of ciliated cells (Figure 1i). These findings indicate that NAD^+^ availability is essential for maintaining normal primary cilia length in this brain region.

### 2.2 SIRT1 Mediates the Regulatory Effect of NAD ^+^ on Primary Cilia

SIRT1 is a major NAD^+^–dependent protein deacetylase involved in the regulation of cellular stress resistance, metabolism, and aging (Imai and Guarente 2014). A previous study has shown that SIRT1 inhibits ciliogenesis by promoting the proteasomal degradation of ciliary proteins in cholangiocinoma cells (Pant et al. 2021). We therefore examined whether SIRT1 functions as a downstream effector in the NAD^+^–mediated regulation of primary cilia in hypothalamic neurons. In contrast to its reported role in cholangiocarcinoma cells (Pant et al. 2021), treatment with the SIRT1 activator resveratrol significantly increased primary cilia length compared with controls in N1 hypothalamic neuron cells (Figure 2a). Similarly, wild type *SIRT1* overexpression markedly extended primary cilia in N1 cells compared with those transfected with an empty vector (Figure 2b), indicating that SIRT1 activation is sufficient to promote cilia elongation in hypothalamic neurons. To determine whether SIRT1–mediated ciliary regulation depends on its deacetylase activity, we examined whether overexpression of the catalytically inactive SIRT1 mutant H363Y promotes primary cilium elongation. Unlike wild–type *SIRT1*, overexpression of *SIRT1 H363Y* failed to increase primary cilium length (Figure 2b). Consistently, a SIRT1 deacetylase activity assay showed that overexpression of wild–type SIRT1 increased SIRT1 deacetylase activity, whereas overexpression of *SIRT1 H363Y* had no effect (Figure S3a). Conversely, treatment with EX–527, a selective SIRT1 inhibitor that suppresses SIRT1 deacetylase activity (Gertz et al. 2013), abolished NR–induced cilium elongation (Figure 2c and Figure S3b). Likewise, small interfering RNA (siRNA)–mediated knockdown of *Sirt1* significantly attenuated NMN–induced cilium elongation and SIRT1 activation (Figure 2d, Figures S3c and S4a). Together, these findings demonstrate that SIRT1 deacetylase activity is required for NAD^+^–dependent regulation of primary cilium length.

**FIGURE 2:**
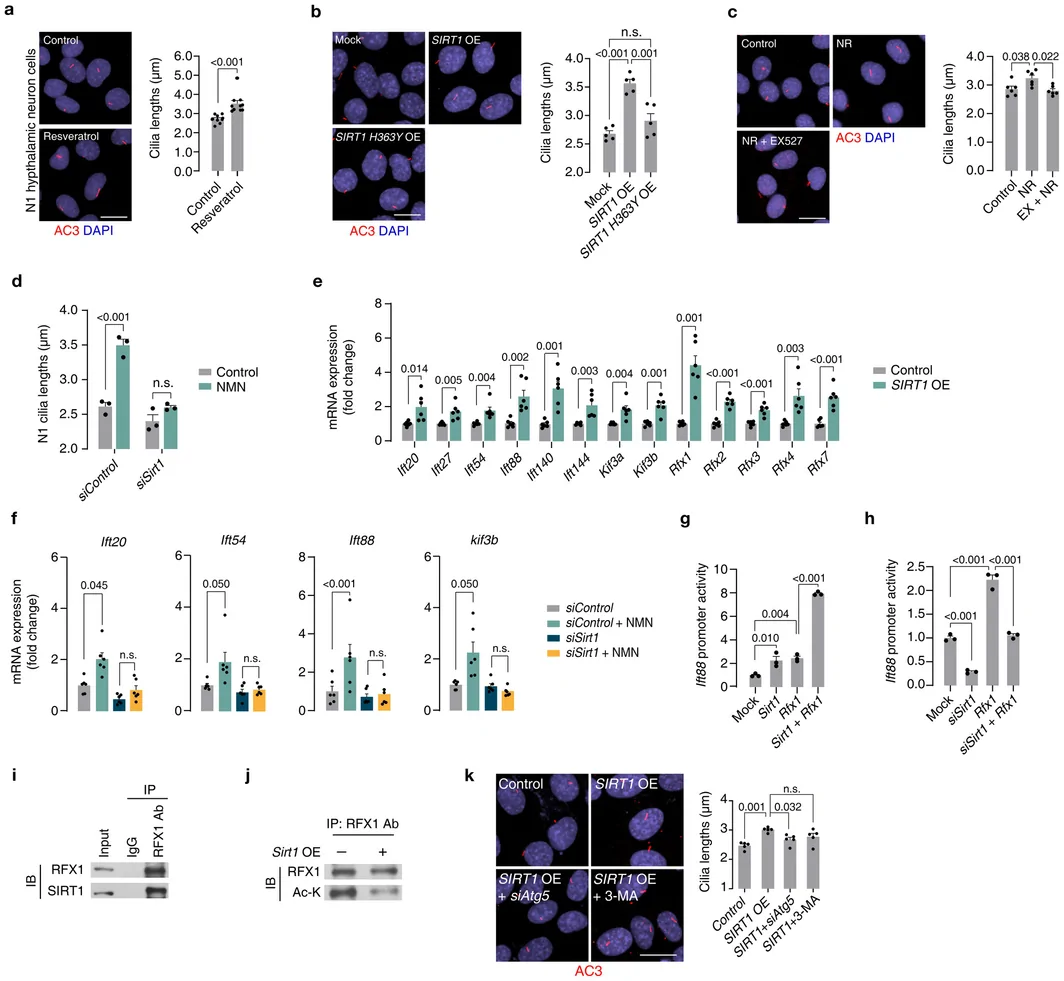
SIRT1 mediates the regulatory effect of NAD^+^ on primary cilia. (a) SIRT1 activator resveratrol (20 μM) treatment elongates cilia length in N1 cells (*n* = 8 wells per group). AC3: Adenylyl cyclase 3. (b) Effects of overexpression (OE) of wild–type *SIRT1* and its catalytically inactive *H363Y* mutant on ciliary length in N1 cells (*n* = 5 wells per group). (c) Effect of treatment with the SIRT1 inhibitor EX527 (100 μm for 16 h) on NR (100 μM)‐induced cilia growth (*n* = 6 wells per group). (d) *Sirt1* knockdown with small inhibitory RNA (*siSirt1*) prevents NMN (100 μM)–induced cilia elongation (*n* = 3 wells per group). (e) Effect of *SIRT1* OE on mRNA expression levels of multiple cilia‐related genes (*n* = 6 wells per group). (f) *Sirt1* knockdown inhibits NMN‐induced upregulation of cilia‐related genes (*n* = 6 wells per group). (g, h) SIRT1 and RFX1 cooperatively regulate the promoter activity of the *Ift88* gene (*n* = 3 wells per group). (i, j) Molecular interaction between SIRT1 and RFX1, and SIRT1‐mediated deacetylation of RFX1, demonstrated by sequential immunoprecipitation (IP) and immunoblotting (IB) in N1 cells with or without *SIRT1* OE. Ac‐K, acetylated lysine. (k) Effect of autophagy inhibition by *Atg5* knockdown or 3‐MA treatment on SIRT1‐induced changes in primary cilia (*n* = 5). Data were obtained from three independent experiments and are presented as the mean ± SEM. Statistical analyses were performed using two‐sided unpaired Student's *t‐*test (a, e), one‐way ANOVA (b, c, f, g, h, k), or two‐way (d) ANOVA with Tukey's post hoc test. n.s., not significant. Scale bars, 20 μm.

Building on the established role of SIRT1 in transcriptional regulation through the deacetylation of transcription factors (Haigis and Sinclair 2010), we next investigated whether modulation of NAD^+^ levels influences the expression of genes essential for cilia growth via SIRT1. In N1 cells, *Sirt1* overexpression significantly increased the mRNA levels of several cilia growth–related genes (Haigis and Sinclair 2010; Taschner et al. 2012), including *Ift20*, *Ift27*, *Ift54*, *Ift88*, *Ift140*, *Ift144*, *Kif3a*, *Kif3b, Rfx*1, *Rfx*2, *Rfx*3, *Rfx*4, and *Rfx*7 (Figure 2e). Consistently, NMN treatment upregulated the expression of *Ift20*, *Ift54*, *Ift88*, and *Kif3a*, and this effect was abolished by *Sirt1* depleted cells (Figure 2f and Figure S4a). These results identify SIRT1 as a key mediator of NAD^+^–dependent ciliary regulation, acting at least in part through transcriptional control of cilia growth–related genes in hypothalamic neurons.

In our previous study, we have showed that leptin promotes ciliary growth in hypothalamic neurons by stimulating the activity of the cilia–related transcription factor RFX1 (Kang et al. 2015). We therefore investigated whether SIRT1 regulates RFX1–mediated transcriptional activation of *Ift88*. *Sirt1* overexpression enhanced RFX1's effect on *Ift88* transcriptional activity, whereas *Sirt1* knockdown significantly suppressed it (Figure 2g,h and Figure S4a). To further investigate whether RFX1 serves as a deacetylation target of SIRT1, we overexpressed *Rfx1* with or without *Sirt1* in N1 cells and performed immunoprecipitation using an RFX1 antibody, followed by immunoblotting for SIRT1, RFX1, and acetylated lysine. We found a molecular interaction between SIRT1 and RFX1 (Figure 2i). In cells overexpressing SIRT1, acetylated RFX1 levels were reduced (Figure 2j). These findings suggest that SIRT1 binds and deacetylates RFX1. Collectively, these results identify SIRT1 as a key mediator of NAD^+^–dependent ciliary regulation, acting at least in part through deacetylation of RFX1 and subsequent transcriptional activation of cilia growth–related genes in hypothalamic neurons.

Meanwhile, SIRT1 has been implicated in the regulation of autophagy (Sacitharan et al. 2020), and autophagy has been linked to ciliogenesis (Pampliega et al. 2013; Tang et al. 2013). We therefore investigated whether SIRT1 promotes ciliary growth through the regulation of autophagy. Knockdown of *Atg5*, which impairs autophagosome formation (Mizushima et al. 2001), abolished SIRT1–induced primary cilium elongation (Figure 2k, Figures S4b and S5a). In contrast, co–treatment with 3–methyladenine (3–MA), an inhibitor of autophagy initiation that blocks class III phosphatidylinositol 3–kinase (PI3K) (Wu et al. 2010), did not significantly affect SIRT1–induced cilium elongation (Figure 2k). These findings suggest that ATG5–dependent mechanisms contribute to SIRT1‐mediated cilium elongation, although the precise contribution of canonical autophagy remains uncertain.

### 2.3 Primary Cilia Are Essential for the NAD ^+^–Mediated Regulation of Lysosomal Activity

Having established that NAD^+^ regulates the architecture of primary cilia via SIRT1, we next asked whether this ciliary remodeling has functional consequences for cellular homeostasis. Primary cilia are increasingly recognized as critical hubs for integrating signaling pathways that govern organelle function, including that of the mitochondria (Bae et al. 2019; Singla and Reiter 2006). Because cilia are closely linked to the regulation of autophagy (Orhon et al. 2015; Pampliega et al. 2013), we hypothesized that NAD^+^–mediated modulation of primary cilia may influence lysosomal function in hypothalamic neurons. To test this hypothesis, we first examined whether alterations in cellular NAD^+^ levels affect lysosomal acidification, a critical determinant of lysosomal degradative activity (DiCiccio and Steinberg 2011), using LysoSensor dyes in N1 hypothalamic neuronal cells subjected to 24 h of serum deprivation before treatment. Treatment with NAD or its precursors, NMN and NR, markedly increased LysoSensor fluorescence intensity compared with untreated controls (Figure 3a). Although serum deprivation is known to increase cellular NAD^+^ levels (Yang et al. 2007), these findings indicate that further elevating NAD^+^ availability enhances lysosomal acidification in serum–deprived hypothalamic neurons. Consistent with the effects of increased NAD^+^ availability, overexpression of wild–type SIRT1 increased LysoSensor fluorescence intensity in N1 hypothalamic neuronal cells, whereas overexpression of the catalytically inactive SIRT1 H363Y mutant had no effect on LysoSensor fluorescence (Figure 3b). These findings indicate that SIRT1 regulates lysosomal acidification in a deacetylase activity–dependent manner. Conversely, depletion of cellular NAD^+^ by either FK866 treatment or *Nampt* knockdown markedly reduced LysoSensor fluorescence intensity (Figure 3c,d, Figures S4c and S5b). Together, these results demonstrate that cellular NAD^+^ availability is essential for the regulation of lysosomal acidity.

**FIGURE 3:**
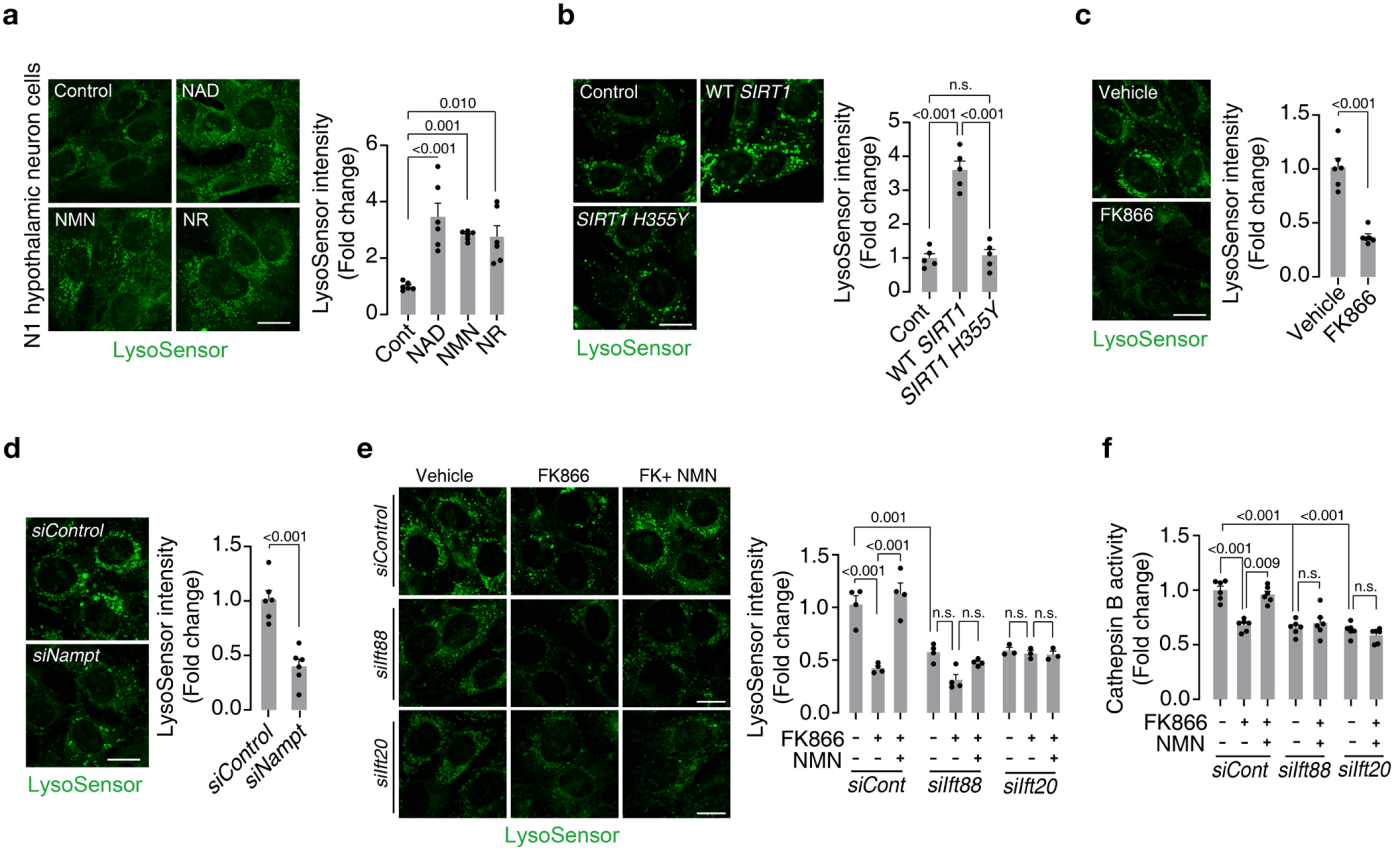
Primary cilia are essential for NAD^+^‐mediated regulation of lysosomal acidity. (a) Effects of NAD^+^ elevation induced by NAD or its precursors (NMN, NR) treatment on lysosomal acidity, assessed by LysoSensor fluorescence intensity (*n* = 6 wells per group). Scale bars, 20 μm. (b) Effect of overexpression of wild‐type *SIRT1* and a catalytically inactive *SIRT1* mutant (*H363Y*) on lysosomal acidity (*n* = 5 wells per group). (c, d) Effects of NAD^+^ depletion induced by FK866 treatment or *Nampt* knockdown on lysosomal acidity (*n* = 6 wells per group). Scale bars, 20 μm. (e) Restorative effects of NMN co‐treatment on the FK866‐induced lysosomal deacidification in N1 cells transfected with control siRNA (*siControl*), *Ift88 siRNA* (*siIft88*), *or Ift20* siRNA (*siIft20*) (*n* = 4 wells per group). Scale bars, 20 μm. (f) Effects of NAD^+^ depletion (FK866) and repletion (FK866 + NMN) on lysosomal cathepsin B activity, assessed by the Magic Red assay, in N1 cells with or without ciliary dysgenesis (*n* = 6 wells per group). Data were obtained from three independent experiments and are presented as the mean ± SEM. Statistical analyses were performed using two‐sided unpaired Student's *t*‐test (c, d), one‐way ANOVA (a, b), or two‐way ANOVA (e, f), followed by Tukey's post hoc test. n.s., not significant.

To determine whether primary cilia mediate NAD^+^–dependent regulation of lysosomal function, we assessed lysosomal acidification following modulation of cellular NAD^+^ levels in cells with impaired ciliogenesis. Primary cilia formation was disrupted by siRNA‐mediated knockdown of *Ift88* or *Ift20* (Figures S4d,e and S5c,d). In cells transfected with nontargeting siRNA, NMN treatment restored the FK866–induced reduction in LysoSensor fluorescence (Figure 3e). In contrast, *Ift88* knockdown alone markedly reduced LysoSensor fluorescence and attenuated both the FK866–induced decrease and the NMN–mediated restoration of lysosomal acidification (Figure S3e). Similarly, knockdown of another ciliary gene, *Ift20*, also attenuated the effects of FK866 and NMN on LysoSensor fluorescence (Figure 3e). These findings indicate that intact primary cilia are required for NAD^+^–dependent regulation of lysosomal acidification. Consistent with these observations, cathepsin B activity, assessed using the Magic Red assay, was reduced by FK866 treatment and restored by NMN co–treatment in control cells. However, the restorative effect of NMN was abolished in *Ift88–* or *Ift20–*deficient cells (Figure 3f). Together, these findings demonstrate that primary cilia are essential mediators of NAD^+^–dependent regulation of lysosomal acidification and lysosomal proteolytic function.

### 2.4 NAD ^+^ Depletion Impairs CHMP4B Shuttling From Periciliary Region to Lysosomes in Response to Lysosomal Stress

Lysosomes preserve their membrane integrity through specialized repair mechanisms (Radulovic et al. 2026). When lysosomal membranes are damaged by oxidative stress or proteotoxic insults, repair pathways, including the endosomal sorting complexes required for transport–III (ESCRT–III), are rapidly recruited to restore lysosomal function (Radulovic et al. 2018). Notably, recent studies have demonstrated that the efficiency of lysosomal repair declines with age, leading to increased lysosomal fragility and cellular vulnerability in aged tissues (Vrancx and Annaert 2025). Interestingly, a previous study reported the localization of charged multivesicular body protein 4B (CHMP4B) within primary cilia (Jung et al. 2020), suggesting a potential cilia–associated role for this ESCRT–III component. We therefore examined whether CHMP4B might serve as a molecular link between primary cilia and lysosomes.

We first sought to verify the localization of CHMP4B in the ciliary structure of hypothalamic neurons by performing double immunostaining with antibodies against CHMP4B and either acetylated tubulin or γ–tubulin, which label the primary cilium and basal body, respectively. In control N1 cells, CHMP4B was abundantly localized near the basal body, with lower expression within the axoneme of primary cilium, indicating that under basal conditions, CHMP4B is predominantly enriched around the basal body (Figure 4a). Remarkably, when cellular NAD^+^ levels were depleted by FK866 treatment, CHMP4B expression in the periciliary region was markedly decreased (Figure 4a,b). This change in localization was reversed by co–treatment with NMN, restoring CHMP4B distribution to a pattern similar to that of control cells (Figure 4a,b). To more precisely determine the localization of CHMP4B at periciliary satellites, we performed co–immunostaining for CHMP4B and pericentriolar material 1 (PCM1), a marker of periciliary satellites (Figure 4a). Quantitative analysis revealed a significant reduction in CHMP4B intensity at PCM1^+^ periciliary satellites in FK866–treated cells compared with controls. This reduction was reversed by NMN treatment, indicating an NAD^+^–dependent presence of CHMP4B in the periciliary satellites (Figure 4c).

**FIGURE 4:**
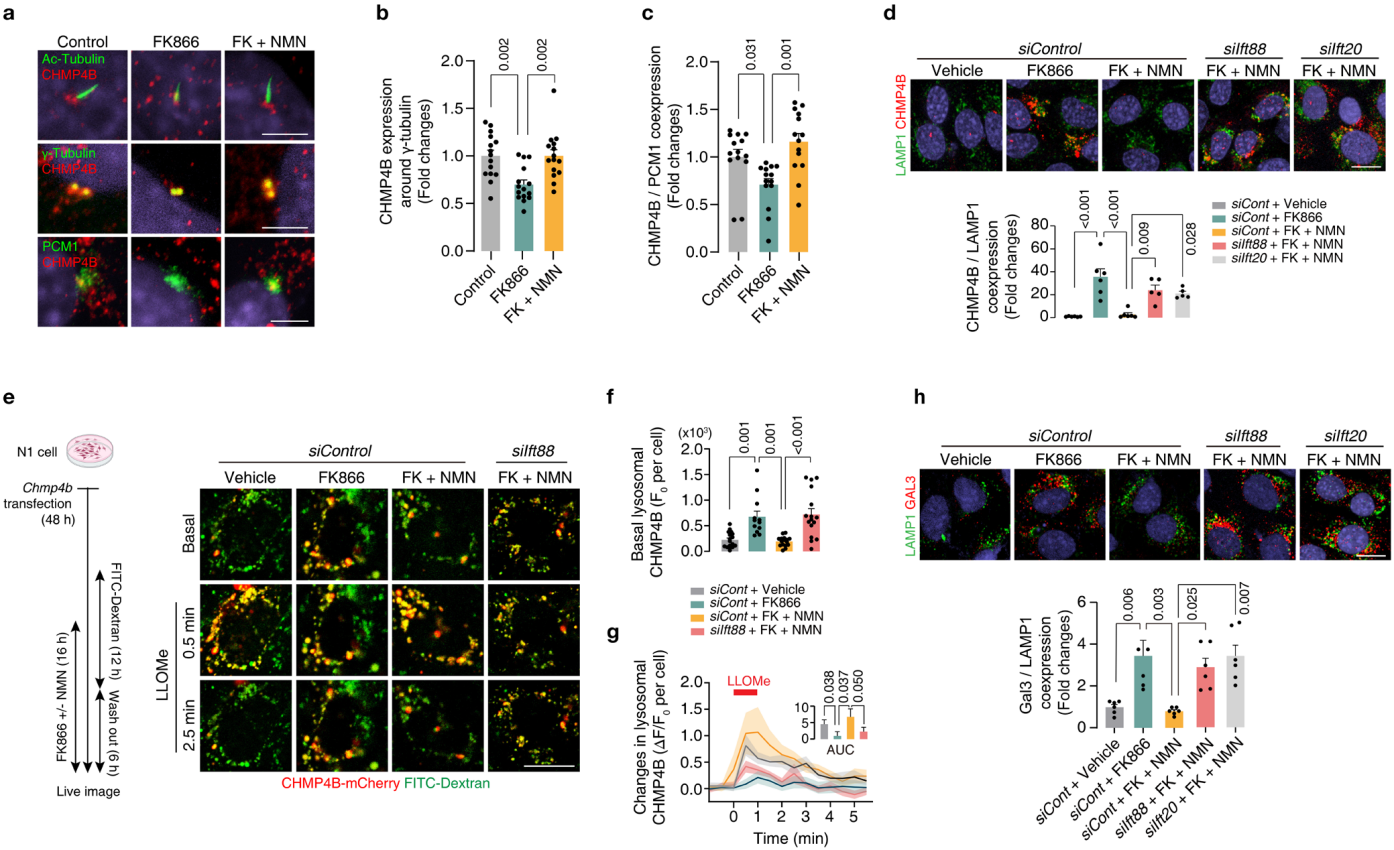
NAD^+^ depletion impairs CHMP4B shuttling from periciliary region to lysosomes in response to lysosomal stress. (a–c) Changes in CHMP4B localization to the cilium (acetylated tubulin), the peribasal body region (γ–tubulin), and the pericentriolar satellite (pericentriolar material 1 [PCM1]) during NAD^+^ depletion (FK866) and repletion (FK866 + NMN) in N1 cells (b, *n* = 15 wells per group; c, *n* = 14 wells per group). Scale bars: 5 μm. (d) Changes in lysosomal CHMP4B localization during NAD^+^ depletion and repletion in N1 cells with or without ciliary defects induced by *Ift88* or *Ift20* knockdown (*n* = 5–6 wells per group). Scale bars, 20 μm. (e–g) Time–lapse imaging (e) and quantification (f, g) of dynamic CHMP4B recruitment to FITC–dextran–labeled lysosomes before (f) and after (g) LLOMe–induced lysosomal stress in N1 cells subjected to NAD^+^ depletion or repletion and transfected with control or *Ift88* siRNAs (*n* = 15–18 cells per group). Scale bars, 20 μm. (h) Lysosomal permeability, assessed by lysosomal Galectin–3 accumulation, in N1 cells subjected to NAD^+^ depletion or repletion and transfected with control or *Ift88 or Ift20* siRNAs (*n* = 5–6 wells per group). Scale bars, 20 μm. Data were obtained from three independent experiments and are presented as the mean ± SEM. Statistical analyses were performed using one–way ANOVA with Tukey's post hoc test (b, c, d, f, g, h).

Given the marked decrease in CHMP4B presence at periciliary sites under NAD^+^–depleted conditions, we hypothesized that CHMP4B may instead traffic to lysosomes under these conditions. To test this, we performed co–immunostaining for CHMP4B and LAMP1 in N1 cells treated with FK866, with or without NMN. Lysosomal localization of CHMP4B was increased following FK866 treatment, and this effect was inhibited by co–treatment with NMN (Figure 4d). Notably, the inhibitory effect of NMN on CHMP4B lysosomal localization was diminished in *Ift88–* and *Ift20–*deficient cells (Figure 4d, Figures S4d,e and S5c,d). Therefore, NAD^+^ depletion may promote the shuttling of CHMP4B from the periciliary region to lysosomes, and this process depends on primary cilia.

We also tested whether cellular NAD^+^ levels affects CHMP4B shutting to lysosomes upon the lysosomotropic dipeptide L–leucyl–L–leucine methyl ester (LLOMe)–induced lysosomal membrane damage. We assessed the dynamics of the recruitment of CHMP4B to the lysosomal membrane by quantifing its colocalization with FITC–dextran, that labels lysosomes, in cells expressing mCherry–tagged CHMP4B (Figure 4e). Upon LLOMe treatment, CHMP4B rapidly accumulated on lysosomal membranes within 1 min, indicating acute membrane damage–induced recruitment (Figure 4e). Interestingly, in FK866–treated cells, CHMP4B intensity on lysosomes was already elevated prior to LLOMe exposure, suggesting that lysosomal stress was present even before acute damage (Figure 4e,f). It also showed minimal further increase after LLOMe treatment (Figure 4e,g). This basal expression level and abnormal recruitment pattern were reversed by NMN co–treatment (Figure 4e–g). *Ift88* knockdown prevented NMN from normalizing basal lysosomal CHMP4B accumulation and LLOMe–induced CHMP4B recruitment dynamics under FK866–treated conditions (Figure 4e–g). These data further support the cilia–dependent regulation of CHMP4B trafficking and suggest that in NAD^+^–depleted cells, further recruitment of CHMP4B to sites of lysosomal membrane injury is impaired, potentially resulting in defective lysosomal membrane repair and increased lysosomal permeability.

To directly assess lysosomal membrane integrity, we examined the colocalization of LAMP1 and galectin–3, as the recruitment of galectin–3 to LAMP1–positive compartments is a well–established marker of lysosomal membrane damage (Aits et al. 2015). FK866 treatment increased the colocalization of galectin–3 and LAMP1 (Figure 4h), indicating enhanced lysosomal membrane damage. This effect was completely reversed by NMN supplementation; however, the protective effect of NMN was markedly attenuated in *Ift88–* and *Ift20*–deficient cells (Figure 4h, Figures S4d,e and S5c,d). These findings suggest that intact primary cilia are required for the NAD^+^–dependent maintenance of lysosomal integrity.

### 2.5 NAD ^+^ Activates Lysosomal CHMP4B–TRPML1–AMPK Signaling Pathway

Lysosomes serve as important intracellular Ca^2+^ stores and actively release Ca^2+^, playing a critical role in cellular signaling pathways (Lloyd–Evans and Waller–Evans 2020). This Ca^2+^ signaling is essential for processes such as autophagy, lysosomal membrane trafficking, and lysosomal function (Lloyd–Evans and Waller–Evans 2020). TRPML1 is a lysosomal membrane Ca^2+^ channel involved in intracellular Ca^2+^ signaling (Lloyd–Evans and Waller–Evans 2020). Given reports that lysosomal lipid accumulation and dysfunction can impair the function of TRPML1 (Grieco et al. 2025; Shen et al. 2012), we investigated how NAD^+^ levels and ciliary changes affect TRPML1 activity. To this end, we monitored changes in GCaMP fluorescence intensity in cells expressing TRPML1–GCaMP, a lysosome–targeted Ca^2+^ sensor (Shen et al. 2012). NMN supplementation enhanced TRPML1–GCaMP fluorescence upon stimulation with ML–SA1, a specific TRPML1 agonist (Figure 5a). In contrast, cilia–deficient cells exhibited a markedly blunted TRPML1–GCaMP response to ML–SA1, which was not restored even after NMN treatment (Figure 5a, Figures S4d,e, S5c,d, and S6). These results imply that NAD^+^ maintains TRPML1 channel activity in a cilia–dependent manner, and that ciliary integrity is important for NAD^+^–mediated activation of lysosomal Ca^2+^ signaling.

**FIGURE 5:**
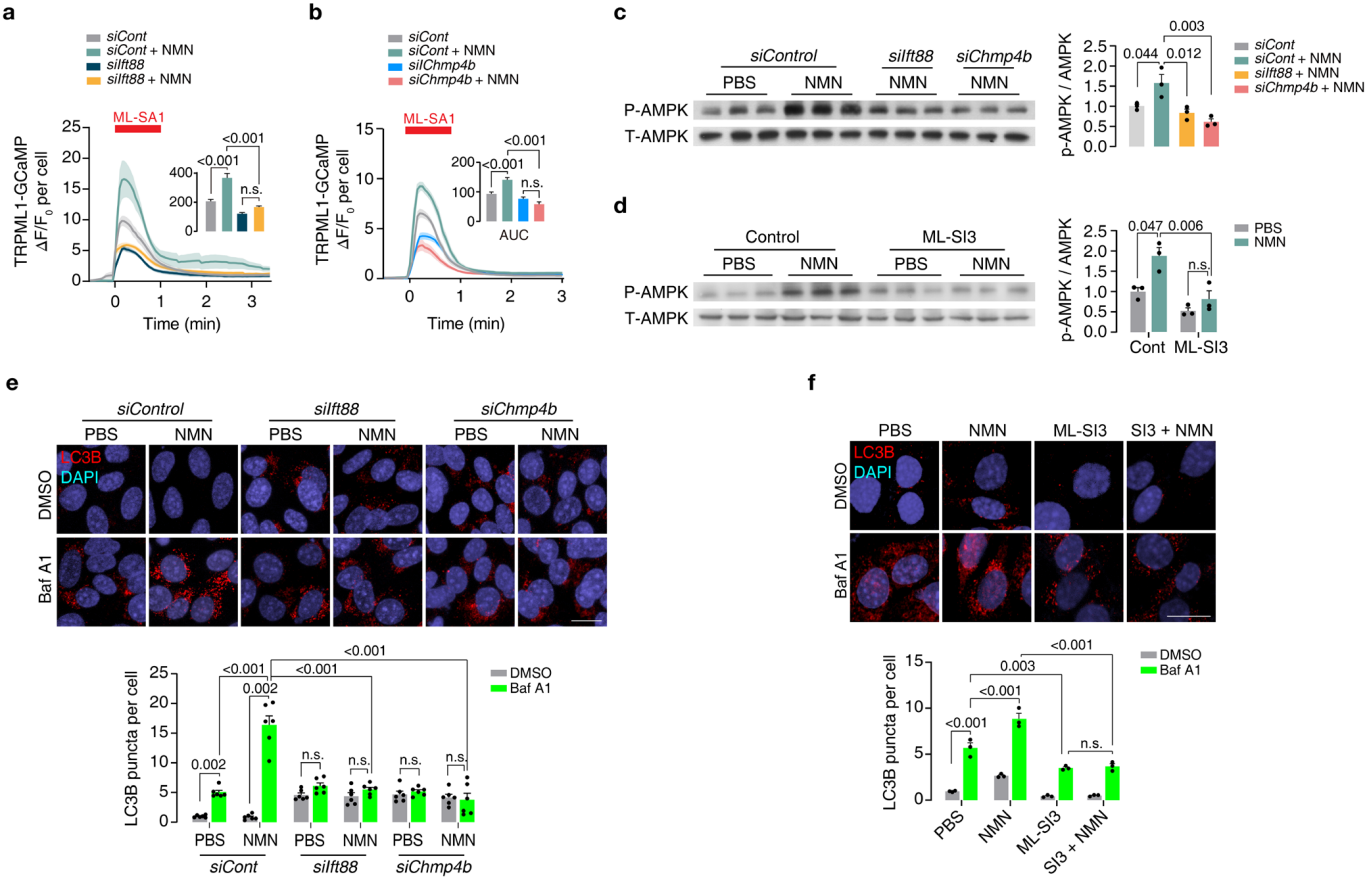
NAD^+^ activates lysosomal CHMP4B–TRPML1–AMPK signaling pathway. (a, b) Effects of NMN on TRPML1 agonist (ML–SA1)–induced lysosomal Ca^2+^ release, assessed by TRPML1–GCaMP intensity, in N1 cells with *Ift88* or *Chmp4b* depletion (a; *n* = 17–22 cells, b; *n* = 22–26 cells per group). (c, d) Effects of *Ift88* or *Chmp4b* depletion or TRPML1 inhibition with ML–SI3 on NMN–induced AMPK activation, assessed by AMPK phosphorylation, in N1 cells (*n* = 3 wells per group). (e, f) Effects of NMN on autophagy flux, assessed by the difference between basal and post–bafilomycin A1 (BafA1) LC3B puncta counts, in N1 cells with *Ift88* or *Chmp4b* depletion or TRPML1 inhibition by ML–SI3 (e; *n* = 6 wells, f; *n* = 3 wells per group). Scale bars, 20 μm. Data were obtained from two or three independent experiments and are presented as the mean ± SEM. Statistical analyses were performed using two–way ANOVA with Tukey's post hoc test (a, b, c, d, e, f). n.s., not significant.

Since CHMP4B recruitment is associated with NAD^+^–dependent ciliary function, we next examined whether CHMP4B is necessary for TRPML1 activation by assessing TRPML1–GCaMP fluorescence in CHMP4B–deficient cells. ML–SA1–induced increase in TRPML1–GCaMP fluorescence in cells with CHMP4B knockdown compared to control cells, and this impairment was not rescued by NMN treatment (Figure 5b and Figure S4f). These results support the notion that the NAD^+^–cilia axis regulates TRPML1 channel activity through CHMP4B. Notably, CHMP4B knockdown also disrupted ciliogenesis (Figure S5e), indicating a bidirectional regulatory relationship between primary cilia and lysosomes.

We next examined whether CHMP4B physically associates with TRPML1 by co–immunoprecipitation. TRPML1 was enriched in mCherry–CHMP4B immunoprecipitates compared with IgG controls, indicating a physical association between CHMP4B and TRPML1 under our experimental conditions (Figure S7). These findings suggest that CHMP4B may regulate TRPML1 channel activity through their molecular association, potentially by facilitating the localization, stabilization, or functional organization of TRPML1 at the lysosomal membrane. Together, these data support a model in which CHMP4B serves as a molecular link between cilia–dependent lysosomal membrane regulation and TRPML1–mediated Ca^2+^ signaling.

We further investigated the downstream signaling events associated with TRPML1 modulation by the NAD^+^–cilia axis. As the cellular energy sensor AMP–activated protein kinase (AMPK) is activated by TRPML1–mediated lysosomal Ca^2+^ signaling (Scotto Rosato et al. 2019), we examined whether NMN treatment can activate AMPK through mechanisms dependent on primary cilia, CHMP4B, and TRPML1. NMN–induced AMPK activation was assessed by measuring AMPK phosphorylation via immunoblotting in N1 cells following *Ift88* or *Chmp4b* depletion, or treatment with the TRPML1 chemical antagonist ML–SI3. NMN treatment led to robust AMPK activation; however, this response was almost completely abolished in *Ift88– or Chmp4b*–depleted cells (Figure 5c, Figures S4d,f and S5c,e). These findings indicate that an intact cilia–lysosome axis is critical for NMN–induced AMPK activation. ML–SI3 treatment also reduced NMN–induced AMPK phosphorylation, suggesting that TRPML1–mediated lysosomal Ca^2+^ release contributes to NMN–induced AMPK activation (Figure 5d).

Since both NAD^+^ and AMPK are known to enhance autophagy processes (Wang et al. 2022; Wilson et al. 2023), we next examined the role for the cilia–CHMP4B–TRPML1 axis on NAD^+^–activated autophagy. In these experiments, autophagy was assessed by staining for LC3B puncta, and autophagic flux was measured by evaluating LC3B puncta accumulation following bafilomycin A1 treatment. NMN treatment significantly increased bafilomycin A1–induced LC3B puncta accumulation (Figure 5e,f). However, this increase was markedly attenuated in cells depleted of *Ift88* or *Chmp4b*, or treated with ML–SI3 (Figure 5e,f), indicating that functional cilia–CHMP4B–TRPML1 axis is essential for promoting autophagy downstream of NAD^+^ signaling. Interestingly, *Ift88*–depleted cells exhibited increased basal LC3 puncta accumulation (Figure 5e). To determine whether this increase resulted from impaired autophagic flux caused by lysosomal dysfunction, we inhibited autophagosome formation by knocking down *Atg5* (Figure S4b). Inhibition of autophagosome formation abolished the basal accumulation of LC3 puncta induced by *Ift88* depletion (Figures S4d and S8). These findings suggest that the increased basal LC3 puncta observed in *Ift88*–depleted cells result from impaired autophagic flux and defective clearance of LC3–positive compartments, rather than enhanced autophagy induction. Collectively, our findings suggest that the cilia–lysosomal axis plays a critical role in modulating lysosomal Ca^2+^ signaling and autophagic activity in response to in intracellular NAD^+^ alterations.

### 2.6 Reversal of NAD ^+^ Depletion by Calorie Restriction Delays Aging–Associated Metabolic Phenotypes through a Hypothalamic Cilia–Dependent Mechanism

Finally, we explored the physiological and therapeutic relevance of our findings in the context of aging. Because hypothalamic neuronal dysfunction is closely linked to organismal aging (Dacks et al. 2013; Zhang et al. 2013), we investigated whether age–associated NAD^+^ depletion, and its restoration, influence hypothalamic ciliary and lysosomal function during aging.

To chronically and stably elevate hypothalamic NAD^+^ levels, 24–month–old mice were subjected to a stepwise calorie–restriction (CR) regimen, in which caloric intake was gradually reduced from 90% to 80% of normal levels over 6 weeks (Figure 6a). We confirmed that CR restored hypothalamic NAD^+^ levels in aged mice to those of 3–month–old young mice (Figure 6b). We further assessed whether the effects of CR are mediated through hypothalamic cilia–related mechanisms. To this end, a cohort of aged mice received bilateral MBH injections of adeno–associated viruses (AAV) expressing *Ift88* shRNA (*shIft88*) and eGFP 2 weeks prior to the initiation of CR, whereas the remaining aged mice and young control mice received AAVs carrying a nontargeting shRNA (*shControl*) and eGFP (Figure 6a). Successful AAV delivery to the MBH was confirmed by eGFP fluorescence at the bilateral injection sites (Figure S9a). In eGFP–positive MBH regions, AAV–*shIft88* markedly disrupted primary cilia length but did not reduce ciliary cell percentages compared with AAV–*shControl* (Figure 6c,d and Figure S9b). Efficient *Ift88* knockdown was further confirmed by reduced *Ift88* mRNA and IFT88 protein expression, as determined by qPCR and western blot analyses, respectively (Figure S9c,d).

**FIGURE 6:**
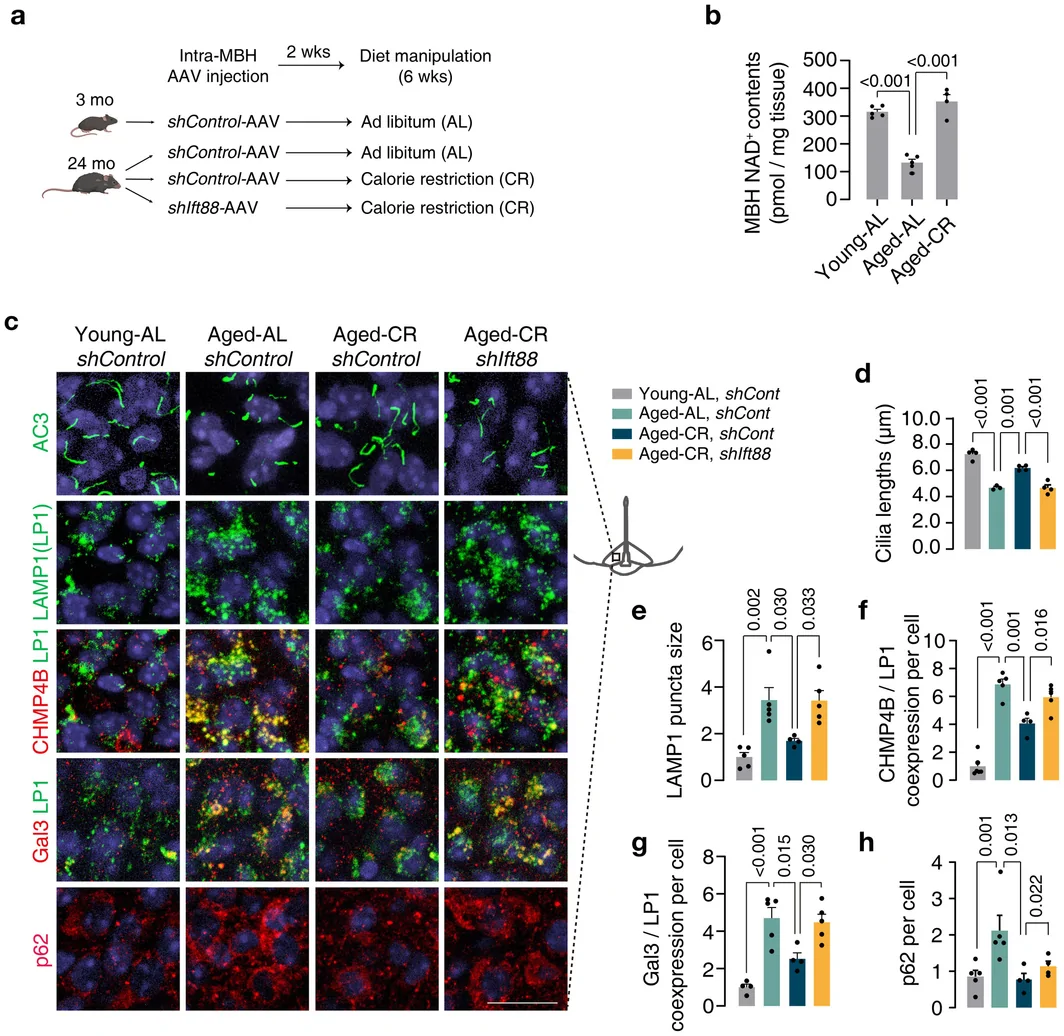
Calorie restriction restores aging–associated reductions in hypothalamic NAD^+^ levels, and ciliary length, and increased lysosomal stress markers in aged mice. (a) Schematic diagram of the study. (b) Comparison of hypothalamic NAD^+^ levels among ad libitum (AL)–fed 3–month–old young mice, 24–month–old aged mice, and calorie–restricted (CR) aged mice (*n* = 4–5 mice per group). (c–h) Comparison of ciliary length (c, d), lysosomal size (c, e), lysosomal CHMP4B levels (c, f), lysosomal membrane permeability, assessed by lysosomal galectin–3 accumulation (c, g), and autophagic activity, assessed by p62 levels (c, h), in the mediobasal hypothalamus (MBH) of young mice, aged mice, calorie–restricted (CR) aged mice, and CR–aged mice with hypothalamic ciliary defects (*n* = 4–6 mice per group). Scale bars, 20 μm. This experiment was performed using one aging cohort, and each dot represents one biologically independent mouse. Data are presented as the mean ± SEM. Statistical analyses used were one–way ANOVA with Tukey's post hoc test (b, d, e, f, g, h).

Lysosomal stress was evaluated by assessing lysosomal enlargement, increased lysosomal CHMP4B expression, and elevated lysosomal membrane permeability, as indicated by galectin–3 localization. Autophagic activity was assessed by measuring the levels of the selective autophagy receptor p62 (Ciuffa et al. 2015), which is degraded through autophagy. Compared to 3–month–old young mice, 24–month–old aged mice exhibited shortened primary cilia accompanied by lysosomal stress in the MBH (Figure 6c–g). Consistent with this lysosomal stress phenotype, increased p62 expression suggested impaired autophagic flux (Figure 6c,h). Notably, CR rescued impaired hypothalamic cilia growth, lysosomal stress and reduced autophagy in the aging hypothalamus (Figure 6c–h). However, these beneficial effects of CR on hypothalamic cilia and lysosomes were attenuated in mice injected with *shIft88–*AAV (Figure 6c–h). These results provide in vivo evidence that NAD^+^ repletion by CR can ameliorate aging–related hypothalamic lysosomal stress and dysfunction through mechanisms dependent on primary cilia.

To determine whether hypothalamic NAD^+^ depletion– and repletion–induced changes in ciliary and lysosomal function contribute to aging–associated metabolic phenotypes, we performed comprehensive metabolic phenotyping, including assessments of body weight, glucose tolerance, and muscle function. Compared with young mice, aged mice fed ad libitum exhibited increased body weight, impaired glucose tolerance, and reduced grip strength (Figure 7a–c). CR in aged mice significantly reduced body weight, improved glucose tolerance, and enhanced grip strength compared with ad libitum–fed aged controls (Figure 7a–c). Notably, these beneficial effects of CR were markedly attenuated by hypothalamic *Ift88* knockdown (Figure 7a–c), indicating that hypothalamic primary cilia are required for the CR–induced improvements in aging–associated metabolic phenotypes.

**FIGURE 7:**
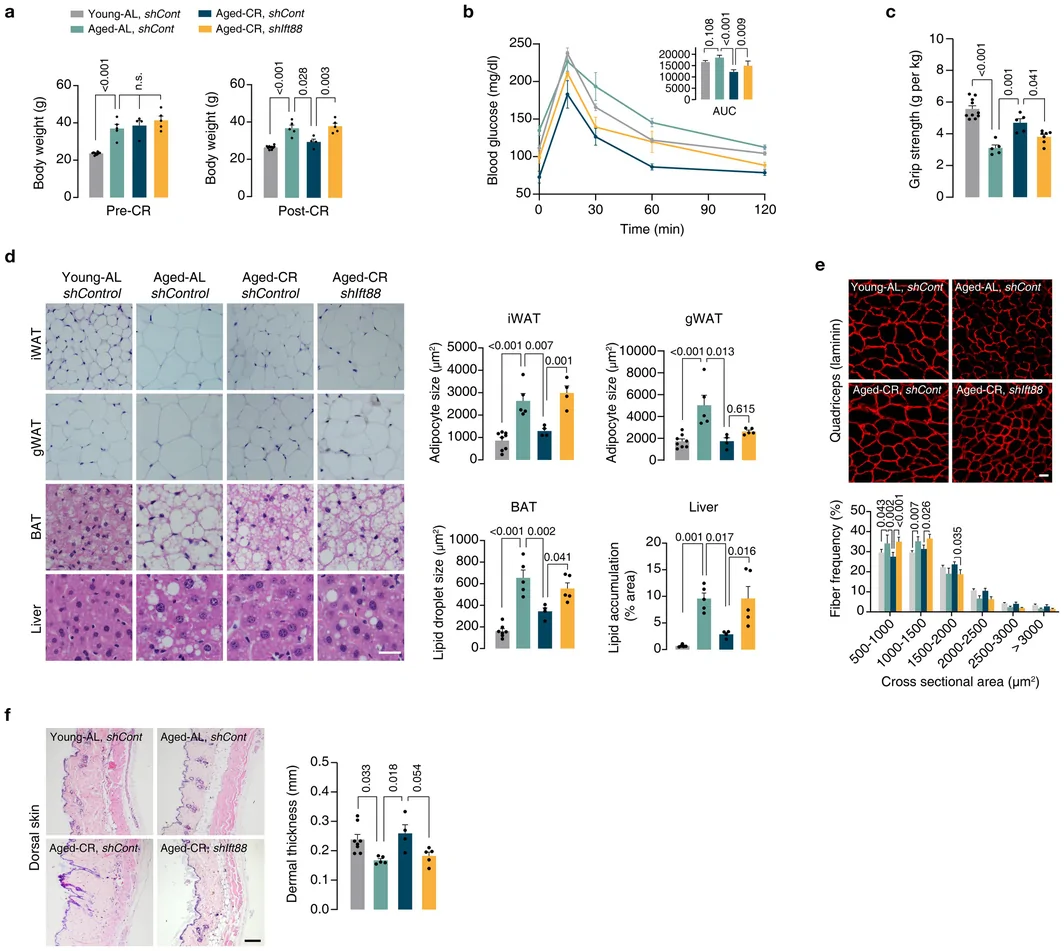
Reversal of NAD^+^ depletion by calorie restriction delays aging–associated metabolic phenotypes in a hypothalamic cilia–dependent mechanism. (a) Body weights before and after calorie restriction (CR) (*n* = 4–10 mice per group). (b, c) Comparison of glucose tolerance and four–limb grip strength among young mice, aged mice, calorie–restricted (CR) aged mice, and CR–aged mice with hypothalamic ciliary defects (*n* = 5–10 mice per group). (d) Histological analysis of inguinal and gonadal white adipose tissue (iWAT, gWAT), brown adipose tissue (BAT), and liver (*n* = 4–8 mice per group). Scale bar, 50 μm. (e, f) Histological analysis of quadriceps muscle and dorsal skin (*n* = 4–8 per group). Scale bar, 100 μm. This experiment was performed using one aging cohort, and each dot represents one biologically independent mouse. Data are presented as the mean ± SEM. Statistical analyses were performed using one–way ANOVA (a–f) and two–way ANOVA (e) with Tukey's post hoc test. n.s., not significant.

Histological analyses of metabolic tissues revealed that aged mice exhibited enlarged adipocytes in inguinal and gonadal white adipose tissue (WAT), as well as increased lipid accumulation in brown adipose tissue (BAT) and the liver, compared with young mice (Figure 7d). CR markedly reversed these age–associated histological alterations, whereas hypothalamic *Ift88* knockdown significantly attenuated the beneficial effects of CR in inguinal WAT, BAT, and the liver (Figure 7d). CR also ameliorated age–related skeletal muscle fiber atrophy and dermal thinning in a hypothalamic cilia–dependent manner (Figure 7e,f). Collectively, these findings suggest that hypothalamic NAD^+^ depletion contributes to aging–associated metabolic dysfunction by disrupting the hypothalamic cilia–lysosome signaling axis, and that restoration of this axis may mitigate these age–related abnormalities.

## 3 Discussion

Metabolic cues are increasingly recognized as critical regulators of organelle structure and function (Gordaliza‐Alaguero et al. 2019). Here, we identify NAD^+^ as a key determinant of primary cilia length in hypothalamic neurons. Both age–related and pharmacologically induced NAD^+^ depletion resulted in ciliary shortening in the MBH neurons, uncovering a previously unrecognized NAD^+^–cilia regulatory axis. Importantly, we demonstrate that NAD^+^ deficiency–induced ciliary dysfunction compromises lysosomal membrane stability and disrupts lysosomal Ca^2+^ signaling via CHMP4B– and TRPML1–dependent pathways. These findings reveal a novel metabolic–signaling axis in which NAD^+^ availability governs lysosomal remodeling through modulation of primary cilia, providing a mechanistic framework for the metabolic regulation of cellular clearance systems. Furthermore, restoration of NAD^+^ levels elongates primary cilia and enhances lysosomal function both in vitro and in vivo. In aged mice, hypothalamic NAD^+^ repletion via CR restores ciliary structure and alleviates lysosomal stress, thereby attenuating aging–associated metabolic phenotypes. Collectively, these results establish the reversibility of NAD^+^–dependent ciliary and lysosomal impairments and position the hypothalamic NAD^+^–cilia–lysosome axis as a potential therapeutic target for age–associated metabolic dysfunction.

While the structural and signaling functions of primary cilia are well recognized (Singla and Reiter 2006), the extent to which their architecture is regulated by cellular metabolic state has begun to be revealed (Han et al. 2014; Pampliega et al. 2013). NAD^+^, a central metabolic cofactor, plays critical roles in mitochondrial function, oxidative stress response, and autophagy, all of which are tightly linked to organelle homeostasis (Imai and Guarente 2014; Verdin 2015). Furthermore, we previously demonstrated that mitochondrial stress promotes cilia elongation in neuronal cells as an adaptive response to cellular stress (Bae et al. 2019). In line with these, our data from both in vivo and in vitro experiments demonstrate that NAD^+^ depletion induced by FK866 treatment results in significant shortening of primary cilia in hypothalamic neurons. Consistently, supplementation with the NAD^+^ precursors NMN and NR further elongated cilia in serum–starved hypothalamic neurons. Together, these findings suggest that NAD^+^ availability may act as a key upstream regulator of ciliary architecture. We propose that the NAD^+^–dependent deacetylase SIRT1 serves as a potential mediator linking NAD^+^ metabolism to ciliary structural dynamics. Our study demonstrated that SIRT1 stimulates the transcription of multiple ciliary genes, at least in part, via deacetylation and activation of the transcription factor RFX1. In contrast to our findings, in cholangiocarcinoma cells, SIRT1 induces the proteasomal degradation of ciliary proteins, including α–tubulin, ARL13B, and KIF3A, leading to ciliary loss and enhanced cell proliferation (Pant et al. 2021). Therefore, the role of SIRT1 in regulating primary cilia may vary depending on the cell type and physiological context.

Given the important roles of primary cilia in sensing both extracellular and intracellular cues (Singla and Reiter 2006), alterations in ciliary architecture are likely to exert downstream effects on other organelles. Notably, the spatial enrichment of autophagy–related proteins at the base and axoneme of the cilium suggests that primary cilia may function as structural and signaling hubs for autophagic regulation (Pampliega et al. 2013; Tang et al. 2013). Our data further demonstrate that NAD^+^ depletion–induced ciliary shortening is associated with impaired lysosomal membrane integrity. Mechanistically, our findings implicate CHMP4B as a key downstream effector linking ciliary defects to lysosomal dysfunction. We observed that CHMP4B, a core component of the ESCRT–III complex responsible for lysosomal membrane repair, is localized to periciliary satellites under basal conditions. Upon lysosomal stress, it translocates to lysosomal membranes. However, under NAD^+^–depleted conditions, this translocation is impaired, leading to disrupted lysosomal membrane remodeling during lysosomal stress and increased lysosomal membrane instability.

Similarly, TRPML1, a lysosomal Ca^2+^ channel essential for lysosomal membrane trafficking and autophagic lysosome reformation (Cao et al. 2017; Lloyd–Evans and Waller–Evans 2020), becomes dysfunctional when ciliary architecture is disrupted. In our study, the NAD^+^–cilia axis regulates TRPML1 activity via a CHMP4B–dependent mechanism, suggesting a critical role for this pathway in maintaining lysosomal Ca^2+^ signaling. As a result, autophagic activity was impaired, which may lead to the accumulation of dysfunctional organelles and proteins in the aging hypothalamus, and age‐related hypothalamic dysfunction. Our findings support a model in which the NAD^+^–cilia axis maintains lysosomal homeostasis by regulating CHMP4B translocation and TRPML1 activity. Although we demonstrated a physical association between CHMP4B and TRPML1, the precise mechanism by which cilia–dependent CHMP4B trafficking regulates TRPML1 function remains unclear.

Progressive hypothalamic dysfunction with age has been implicated in systemic aging and may result from impaired autophagy and proteostasis, elevated proinflammatory cytokine production, dysregulated nutrient sensing, stem cell depletion, and epigenetic alterations in hypothalamic neurons (Aman et al. 2021; Cai and Khor 2019; Kim and Choe 2019; Masliukov and Nozdrachev 2021). We propose an additional mechanism for hypothalamic aging involving dysgenesis of hypothalamic neuronal cilia. Consistent with our findings, a recent study demonstrated that the length of MC4R–bearing cilia in PVN neurons is shortened in aged rats and restored by CR (Oya et al. 2024). That study also showed that cilia on MC4R^+^ PVN neurons mediate feeding responses to satiety signals such as leptin and melanocortin, and that ciliary defects in these neurons lead to increased food intake (Oya et al. 2024). Our study extends these findings by demonstrating that NAD^+^ restoration via CR alleviates lysosomal stress especially in MBH neurons through enhanced ciliary growth. As a result, it improves systemic metabolic parameters, including body weight, glucose tolerance, and lipid accumulation in WAT, BAT, and liver.

In addition, CR enhances motor function, increases muscle fiber size, and thickens the dermis, effects that appear to be mediated, at least in part, by hypothalamic cilia–dependent mechanisms. It is important to note that CR is a pleiotropic intervention and is unlikely to exert its effects exclusively through restoration of hypothalamic NAD^+^ levels. Extensive evidence indicates that CR engages multiple evolutionarily conserved nutrient–sensing and stress–response pathways, including insulin/IGF–1 signaling, mTOR, AMPK, sirtuins, inflammatory signaling, autophagy, mitochondrial metabolism, and neuroendocrine regulation (Fontana et al. 2010; Green et al. 2022). Accordingly, although our data demonstrate that CR restores hypothalamic NAD^+^ levels and improves ciliary and lysosomal phenotypes in aged mice, they do not establish hypothalamic NAD^+^ restoration as the primary mediator of the systemic effects of CR. Rather, our findings support a model in which the hypothalamic NAD^+^–cilia–lysosome axis represents one CR–responsive mechanism that contributes, at least in part, to the amelioration of aging–associated metabolic and tissue dysfunction. Other CR‐responsive pathways in the hypothalamus and peripheral tissues are also likely to act in parallel with, or in coordination with, this axis to mediate the broad physiological benefits of CR.

Notably, in hippocampal neurons, primary cilia mediate osteocalcin signaling to promote cognitive function (Rivagorda et al. 2025). In contrast to the shortening of hypothalamic neuronal cilia, aging–related elongation of cilia in this brain region is associated with memory decline (Rivagorda et al. 2025). Therefore, it would be interesting to determine whether elevation of hippocampal NAD^+^ levels through CR correct ciliary defects and improve memory function in aged mice.

In summary, our findings identify primary cilia as a metabolically sensitive structural hub that links NAD^+^ availability to lysosomal homeostasis in hypothalamic neurons, thereby contributing to the regulation of multiple aging–associated metabolic and tissue phenotypes. Modulation of the hypothalamic NAD^+^–cilia–lysosome axis may thus offer a potential strategy to alleviating age–related physiological decline.

## 4 Methods and Materials

### 4.1 Animals

C57BL/6J male mice aged 3 months were purchased from JA Bio (Suwon, Korea), and 24–month–old mice were obtained from the Animal Facility of Aging Science, Korea Basic Science Institute (Gwangju, Korea). All animals were housed under controlled environmental conditions: temperature maintained at 22°C ± 1°C and a 12–h light/dark cycle with lights on from 8:00 a.m. to 8:00 p.m. Mice had ad libitum access to a standard chow diet (12.5% of calories from fat; Cargill Agri Purina, 38057) and water, except during CR experiments. During the 6–week CR period, mice were provided 90% of the amount of food consumed by ad libitum–fed controls, in Week 1, 85% in Week 2, and 80% for the remaining 4 weeks. All animal procedures were approved by the Institutional Animal Care and Use Committee of the Asan Institute for Life Science (2022IF0034).

### 4.2 Cell Culture

N1 hypothalamic neuronal cells were maintained in Dulbecco's modified Eagle's medium (DMEM) containing 10% fetal bovine serum (FBS) and 1% penicillin/streptomycin. Human retinal pigmented epithelial (RPE–1) cells were maintained in DMEM: F12 containing the same supplements. To induce ciliogenesis, the culture medium was replaced with medium without supplements when the cells were ~90% confluent. Cells were then cultured in serum–free medium for 48 h before ciliary analysis.

### 4.3 NAD ^+^ Measurement

NAD^+^ levels were quantified using an NAD^+^/NADH assay kit (BioAssay Systems, #E2ND–100). Homogenized tissue or cell samples were lysed in the kit–provided NAD^+^ extraction buffer and heated at 60°C for 5 min. After centrifugation, 40 μL of the supernatant was used for NAD^+^ determination according to the manufacturer's protocol. Measured NAD^+^ concentrations were normalized to total protein content.

### 4.4 Cilia Staining and Analysis

For staining primary cilia in N1 neuronal cells, cells were fixed with either 4% paraformaldehyde for 15 min at 4°C, followed by incubation with primary antibodies against acetylated α–tubulin (1:1000; Sigma, #T6793) or adenylyl cyclase 3 (AC3; 1:500; Encor Biotechnology, #RPCA–ACIII) at 4°C for 48 h. For neuronal cilia staining in mice, whole brains were collected after cardiac perfusion with 4% paraformaldehyde for 15 min. Coronal brain sections (30 μm) containing the MBH were obtained using a cryostat. Sections were permeabilized in 0.5% Tween–20 in PBS (PBST) for 10 min, blocked with 3% donkey serum in PBS for 1 h, and incubated with an anti–AC3 antibody (1:500, Encor Biotechnology) at room temperature (RT) for 24 h. After washing, cells or sections were incubated with Alexa Fluor–conjugated secondary antibodies (1:1000; Invitrogen) for 1 h at RT. Nuclei were counterstained with DAPI (1:10,000; Sigma, #9564) for 10 min prior to mounting. Immunofluorescence images were acquired using a confocal microscope (Carl Zeiss LSM 710 or 780), and cilia images were obtained by Z–stacking. The number and length of hypothalamic neuronal cilia were quantified in 3 coronal brain sections per mouse using ZEN software (version 3.1, Carl Zeiss). For visualization purposes, display settings, including white balance, brightness, and contrast, were adjusted uniformly across all AC3 channel images to ensure that ciliary signals were clearly visible and comparable. Individual cilia were measured using the Measure tool in ZEN software. For each cilium, a line was manually traced along its longitudinal axis from base to tip, and the calibrated ciliary length was automatically calculated by the software. These display adjustments were applied solely for image visualization and did not affect the calibrated length measurements (Figure S10). The average ciliary length of approximately 100 MBH neurons per mouse was measured. For cultured neurons, cilia from randomly selected fields (≥ 100 cilia per well) were analyzed. The percentage of ciliated cells was calculated as the number of cilia divided by the number of DAPI–stained nuclei in selected fields. Each treatment was performed in duplicate or triplicate and repeated in at least three independent experiments.

### 4.5 Lysosomal Structure and Functional Analysis

For lysosomal membrane staining in N1 cells, cells were fixed in 4% paraformaldehyde at 4°C for 15 min, permeabilized with 0.01% PBST, and blocked with 3% bovine serum albumin (BSA) in PBS for 1 h. A primary antibody against LAMP1 (1:1000; BD Pharmingen, #553792) was applied and incubated overnight at 4°C. After washing, cells were incubated with Alexa Fluor–conjugated secondary antibodies (1:1000; Invitrogen) for 1 h at RT. To assess lysosomal pH, live N1 cells were incubated with LysoSensor (1:1000; Invitrogen, #7235) for 1 h at 37°C prior to imaging. Cathepsin B activity in live cells was measured using the Magic Red assay (Abcam, #ab270772), following the manufacturer's instructions. Briefly, N1 cells were seeded at 0.5 × 10^4^ cells per well in a 96–well plate and cultured for 24–48 h. The culture medium was then replaced with serum–free medium and cells were incubated for an additional 24 h. Magic Red reagent (Biomol GmbH, #938) was diluted according to the protocol and added to each well (100 μL per well), followed by incubation at 37°C for 1 h. Red fluorescence intensity was subsequently measured. Lysosomal membrane permeability was assessed by examining galectin–3 localization to lysosomes. For this, cells or hypothalamic slices were co–stained with antibodies against galectin–3 and LAMP1. Using the same fixation, permeabilization, and blocking conditions as described above for LAMP1 staining, samples were incubated with a primary antibody mixture containing anti–LAMP1 and anti–galectin–3 (1:1000; Abcam, #ab2785) at 4°C for 48 h. Fluorescence intensity quantification per cell was performed using ImageJ (NIH, version 1.52p) and Photoshop 2025 (Adobe Systems, version 26.3.0).

### 4.6 Lysosomal Ca^2+^ Imaging

For lysosomal Ca^2+^ imaging, N1 cells were cotransfected with a CMV–TRPML1–GCaMP3 plasmid (1 μg), cloned from TRPML1–MYC plasmid (Addgene #18824) at Korea Advanced Institute of Science and Technology, and either nontargeting siRNA or siRNAs targeting *Ift88, Ift20*, or *Chmp4b* (100 pmol) using Lipofectamine 3000 (Invitrogen, #2047113) 48 h before analysis. Cells were treated with or without NMN (100 μM) for 3 h following 16 h of serum starvation prior to imaging. Thirty minutes before imaging, the culture medium was replaced with calcium– and phenol red‐free Hank's Balanced Salt Solution (HBSS; Gibco, #14175). ML–SA1 (a TRPML1 agonist; final concentration 20 μM, Sigma, #SML0627) was added for 1 min to stimulate TRPML1–mediated Ca^2+^ release. Live–cell imaging was performed using a Zeiss LSM 710 confocal microscope (Carl Zeiss, Germany) with a 63× oil immersion objective at 37°C. Images were acquired at 10 s intervals for a total of 9 min. Fluorescence intensity of TRPML1–GCaMP1 was quantified over time using ImageJ (NIH) or Zen software (Carl Zeiss). Regions of interest (ROIs) were drawn around individual lysosomes showing GCaMP1 signal. Changes in background subtracted fluorescence intensity (F) were normalized to baseline (*F*_0_, average of initial 3 frames) and expressed as Δ*F*/*F*_0_. For statistical analysis, the area under the curve (AUC) of Δ*F*/*F*_0_ during the imaging period was calculated and compared across groups.

### 4.7 Immunostaining for CHMP4B Localization

To examine the subcellular localization of CHMP4B in periciliary structures and lysosomes, N1 cells were co–immunostained with antibodies against CHMP4B together with organelle–specific markers, including acetylated α–tubulin (cilia), γ–tubulin (basal bodies), pericentriolar material 1 (PCM1; pericentriolar satellites), and LAMP1 (lysosomes). Except for γ–tubulin staining, which required methanol fixation (−20°C, 4 min), all other immunostaining procedures used 4% paraformaldehyde fixation (4°C, 15 min). Cells fixed in paraformaldehyde were permeabilized with 0.05% PBST and blocked with 3% BSA before incubation with primary antibodies against CHMP4B (1:500; ABclonal, #A7402), acetylated α–tubulin (1:500; Sigma, #T6793), γ–tubulin (1:1000; Invitrogen, #MA119421), PCM1 (1:500; Santa Cruz, #sc–398365), or LAMP1 (1:1000; BD Pharmingen, #553792) at 4°C for 48 h. After washing, appropriate fluorescent dye–conjugated secondary antibodies were applied, and samples were visualized by confocal microscopy.

### 4.8 Live–Cell Imaging of CHMP4B Lysosomal Translocation

To monitor dynamic lysosomal translocation of CHMP4B in response to lysosomal stress, N1 cells were transfected with pLNCX2–mCherry–CHMP4B (Addgene, #116923). To label lysosomes, FITC–dextran (250 μg/mL per confocal dish; Thermo Fisher Scientific, #1844) was added 12 h prior to imaging. Lysosomal stress was induced by treating cells with L–leucyl–L–leucine methyl ester (LLOMe, 0.5 mM; Sigma, #L7393) for 1 min and subsequently washed with PBS. Live–cell imaging was performed using a Zeiss LSM 710 confocal microscope (Carl Zeiss, Germany) equipped with a 63× oil immersion objective at 37°C. Images were captured at 10 s intervals for a total of 9 min. Live–cell imaging procedures followed those described in the Lysosomal Ca^2+^ Signaling section.

### 4.9 Autophagy Analysis

Autophagosomes were visualized by LC3B immunostaining. Autophagic flux was assessed by comparing LC3B puncta accumulation in N1 cells treated with or without bafilomycin A1 (10 nM for 12 h). For LC3B staining, N1 cells were fixed in methanol at −20°C for 4 min, blocked with 3% BSA in PBS, and incubated with anti–LC3B (1:1000, Sigma, #L7543) diluted in blocking solution overnight at 4°C. In the mouse hypothalamus, autophagy was evaluated by p62 immunostaining. For this, hypothalamic slices were rinsed briefly in PBS, permeabilized with 0.5% PBST for 5 min at RT, blocked with 5% donkey serum in PBS for 1 h at RT, and incubated with anti–p62 (1:1000, Abcam, #ab915626) overnight at 4°C. To inhibit autophagy, cells were treated with 3–methyladenine (5 mM for 6 h) or subjected to *Atg5* knockdown.

### 4.10 RNA Silencing

Cells were transfected with 100 pmol of either control siRNA or siRNAs targeting murine *Ift88* (Thermo Fisher, #S75083), *Ift20* (Thermo Fisher, #S79931), *Sirt1* (Thermo Fisher, #S96764), *Chmp4b* (Thermo Fisher, #S93709), or *Atg5* (Thermo Fisher, #S18158) using Lipofectamine 3000 (Invitrogen, #2047113). Forty eight hours later, knockdown efficiency was confirmed by quantitative real–time PCR (Figure S4).

### 4.11 mRNA Quantification

Total RNA was isolated from cells using TRIzol reagent (Invitrogen, #15596018) following the manufacturer's instructions. One microgram of RNA was then reverse–transcribed into cDNA using M–MLV reverse transcriptase (Invitrogen, #28025013). Quantitative real–time PCR was performed on a QuantStudio 5 system (Applied Biosystems) to quantify mRNA expression levels using specific primers (Table S1). mRNA expression levels were normalized to glyceraldehyde 3–phosphate dehydrogenase (*Gapdh*) or β–actin.

### 4.12 Promotor Assay

N1 cells were cultured in 24–well plates and transfected with *Ift88–*luciferase reporter plasmids (pGL3–Ift88, 50 ng, Promega) together with a CMV–β–galactosidase plasmid (25 ng) as an internal control, using Lipofectamine 3000. Cotransfections were performed with or without expression plasmids for *SIRT1* (Flag–SIRT1, 500 ng, Addgene #1791), *Rfx1* (Psg5–Rfx1, 50 ng, provided by Dr. Hsu, National Health Research Institutes, Taiwan), or *siSirt1* (100 pmol), as indicated. After 24–48 h, luciferase activity was measured using a luciferase reporter assay (Promega, #E1501), and values were normalized to β–galactosidase activity to adjust for transfection efficiency. Results are presented as fold change relative to the mock plasmid–transfected control group. All transfections were performed in triplicate and repeated independently at least three times.

### 4.13 Immunoblotting and Immunoprecipitation

Forty microgram of cell lysates were separated using 10% SDS–PAGE and transferred to PVDF membranes (Bio–Rad). After incubation in the blocking buffer (5% BSA in 0.05% PBST), the membranes were incubated overnight at 4°C with antibodies against total AMPK (1:1000, Cell Signaling, #2532s) and phosphorylated AMPK (1:1000, Cell Signaling, #2531). Blots were developed using a horseradish peroxidase (HRP)–conjugated secondary antibody (1:1000) and a chemiluminescent detection system (Thermo Scientific, #32106). Band intensities were quantified using ImageJ software. For validation of AAV–mediated *Ift88* knockdown, MBH tissues containing the eGFP–positive injection sites were collected and processed for immunoblotting as described above. Membranes were incubated with antibodies against IFT88 (1:1000; Proteintech, #13967–1–AP) and β–actin (1:1000; Santa Cruz, #sc–47778). IFT88 protein levels were quantified by densitometry and normalized to β–actin. For the immunoprecipitation assay, N1 cells were grown in a 100 mm culture dish and plated, transfected with an *Rfx1–*expressing plasmid (1 μg) with or without a *SIRT1*–expressing plasmid (1 μg) followed by MG132 (10 μM) treatment. After 48 h, 500 μg of protein extract was obtained and incubated overnight with 4 μg of primary antibody against RFX1 (1:1000; Santa Cruz, #sc–374270) or an equivalent amount of rabbit IgG, followed by incubation with 100 μL of agarose beads (Roche, #10180790). The immunoprecipitates were then subjected to immunoblotting using antibodies specific for RFX1 (1:1000), SIRT1 (1:1000; Cell Signaling, #8469S), and acetylated lysine (1:500; Santa Cruz, #sc–32268). For the CHMP4B–TRPML1 interaction assay, N1 cells were co–transfected with *mCherry–CHMP4B* and *TRPML1–MYC* plasmids (10 μg each), and immunoprecipitation was performed as described above using an anti–CHMP4B antibody (1 μg; ABclonal, #A7402). Immunoprecipitates were analyzed by immunoblotting with antibodies against CHMP4B (1:1000; ABclonal, #A7402) and TRPML1 (1:1000; Invitrogen, #PA1–46474).

### 4.14 SIRT1 Deacetylase Activity Assay

SIRT1 deacetylase activity was measured using a fluorometric SIRT1 activity assay kit (Abcam, #ab156065) according to the manufacturer's instructions. N1 cells were transfected with wild–type *SIRT1* (1 μg; Addgene, #1791), the catalytically inactive *SIRT1 H363Y* mutant (1 μg; Addgene, #1792), or Sirt1–targeting siRNA (100 pmol) for 48 h, or treated with the SIRT1 inhibitor EX527 (100 μM) for 16 h. Equal amounts of cell lysates were incubated with the fluorogenic acetylated substrate, NAD^+^, and the developer provided in the kit. Fluorescence was measured every 1–2 min for 30–60 min using a microplate reader with excitation and emission wavelengths of 350 and 460 nm, respectively. SIRT1 deacetylase activity was determined from the reaction rate during the linear phase of the fluorescence increase and normalized to the control group.

### 4.15 Cell Viability and Cellular Stress Analysis

To determine whether FK866 treatment induced nonspecific cytotoxicity or cellular stress, N1 cells were treated with FK866 (10 nM) for 16 h. Cell viability was assessed using an MTT assay kit (Abcam, #ab211091) according to the manufacturer's instructions. Absorbance was measured at 570 nm using a microplate reader. Apoptosis and cellular stress were evaluated by immunoblotting for cleaved caspase–3 and C/EBP homologous protein (CHOP), respectively. Protein extraction and immunoblotting were performed as described in the Immunoblotting and Immunoprecipitation section using antibodies against caspase–3 (1:1000; Cell Signaling Technology, #9662), cleaved caspase–3 (1:1000; Cell Signaling Technology, #9661), and CHOP (1:1000; Cell Signaling Technology, #2895).

### 4.16 Intracerebroventricular Cannulation and Drug Administration

Stainless steel cannulas (26 gauge; Plastics One) were implanted into the third ventricle of 8–week–old C57BL/6J mice. Stereotaxic coordinates for implantation were 1.5 mm posterior to bregma, at the midline over the sagittal sinus, and 5.0 mm ventral to the skull surface. Surgeries were performed under anesthesia using Zoletil (40 mg/kg) and Rompun (5 mg/kg). One week after surgery, successful cannulation was confirmed by a dipsogenic response to angiotensin II (50 ng). Mice with verified cannula placement then received daily intracerebroventricular injections of FK866 (1 μg in 2 μL of 0.9% saline), with or without NMN (300 μg in 2 μL of 0.9% saline), for five consecutive days using a microsyringe connected to a Hamilton syringe pump. All mice were maintained on a standard chow diet throughout the experiment.

### 4.17 Hypothalamic Virus Injection

Adeno–associated viruses (AAVs) expressing shRNA targeting mouse *Ift88* (AAV–EF1a–DIO–TATAlox–U6–*shIft88*, *shIft88*–AAV), the control virus (AAV–DIO–*shControl*–eGFP; *shControl*–AAV), and AAV–Syn–Cre–eGFP (Syn–Cre–AAV) were generated by the Virus Core Facility at the Korea Institute for Science and Technology. Viral constructs (1 × 10^13^ genome copies/mL) were bilaterally injected into the MBH of 3–month–old or 24–month–old male mice. Stereotaxic injections were performed using the following coordinates: 1.6 mm posterior to bregma, 0.2 mm lateral to the midline, and 5.7 mm ventral to the skull surface. On each side, 0.5 μL of a 1:1 mixture of viral solutions (*shIft88*–AAV or *shControl*–AAV with Syn–Cre–AAV) was infused at a rate of 50 nL/min over 10 min using a microsyringe pump (Harvard Apparatus). After injection, the needle was raised by 0.2 mm and held in place for an additional 10 min to prevent backflow. Beginning 2 weeks after injection, mice were maintained under either ad libitum feeding or calorie restriction for 6 weeks before sacrifice. Successful induction of ciliary dysgenesis was confirmed by reduced cilia length and reduced *Ift88* expression in the MBH at the end of study.

### 4.18 Metabolic Phenotype Analysis

Body weights were monitored weekly during the early light period. For oral glucose tolerance test, D–glucose (1 g/kg, Sigma) was administered via oral gavage in overnight‐fasted mice. Blood was obtained from tail vein for glucose measurement immediately before, and 15, 30, 60, and 120 min after glucose injection. Glucose levels were measured using a glucometer (ACCU–CHEK, Aviva Plus System). Forelimb grip strength was measured using a grip strength meter (BIO–GS3, Bioseb). Mice were held by the tail and gently lowered toward a horizontal metal grid attached to a force transducer. Once the mouse grasped the grid with its forepaws, it was steadily pulled backward in the horizontal plane until it released the grid. The peak force exerted before release was automatically recorded in grams. The test was performed twice, and the average value was used for analysis.

### 4.19 Histological Analysis

Epididymal (gonadal) and inguinal white adipose tissue (WAT), brown adipose tissue (BAT), liver, quadriceps muscle, and skin were dissected after cardiac perfusion with 4% paraformaldehyde for 15 min and then post–fixed in 4% paraformaldehyde overnight. Tissues were then rinsed with distilled water, paraffin–embedded, sectioned at 5 μm thickness, and stained with hematoxylin and eosin (H&E). Lipid droplet area was quantified using ImageJ software (NIH). H&E images were converted to 8–bit grayscale and thresholded to select unstained lipid vacuoles. For each animal, four nonoverlapping fields were randomly selected and all lipid droplets within each field were measured. The mean droplet area per field was calculated and averaged to obtain a single representative value per animal. Muscle fiber analysis was performed by laminin immunostaining. Cryosectioned muscle tissue (20 μm) was incubated in 10% goat serum in 1% PBST at RT for 1 h, followed by incubation with anti–laminin antibody (1:500; Sigma, #L939) overnight at RT. Sections were washed in PBS for 10 min three times and incubated with the appropriate Alexa Fluor–conjugated secondary antibody (1:1000) prior to mounting. Myofiber cross–sectional area (CSA) was quantified using MyoSoft, an ImageJ/Fiji–based macro that employs machine–learning classifiers to segment laminin–positive fiber boundaries, as previously described (Encarnacion‐Rivera et al. 2020).

### 4.20 Statistical Analysis

The statistical analysis was performed using Prism software version 8.0 (GraphPad) or SPSS version 24 (IBM Analytics). Statistical significance among the groups was assessed using one–way or two–way ANOVA followed by Tukey's LSD post hoc analysis or a two–sided unpaired Student's *t*–test, as appropriate. Statistical significance was defined at *p* < 0.05. The data are presented as mean ± standard error of the mean (SEM).

## Author Contributions

S.E.P., E.R., and M.‐S.K. designed the study. S.E.P., E.R., J.W.P., S.J.K., S.Y.J., C.B.P., H.S.L., J.S.K., M.G.K., M.J.K., Y.I.C., W.J., and G.M.K. performed the experiments and/or analyzed the data. S.‐Y.K., and S.H.M., discussed the data. S.E.P., and M.‐S.K. wrote the manuscript. All authors read and edited the manuscript and approved the final version.

## Funding

This study was supported by grants from the National Research Foundation of Korea (NRF) funded by the Ministry of Science and ICT of Korea (RS‐2026‐25526181, RS‐2025‐00518898, RS‐2024‐00393728, RS‐2024‐00406845, RS‐2022‐NR072336), the Korea Health Technology R&D Project through the Korea Health Industry Development Institute (KHIDI) (RS‐2024‐00438349), the Ministry of Health & Welfare, Republic of Korea (RS‐2024‐00404132).

## Conflicts of Interest

The authors declare no conflicts of interest.

## Supporting information


**Figure S1:** FK866 treatment does not induce cell death or cellular stress in N1 cells. Cellular viability was assessed by using MTT assay after treatment with FK866 (10 nM, 16 h) in N1 cells (*n* = 5 wells per group). Data are presented as mean ± SEM. Statistical analyses were performed using two‐sided unpaired Student's *t*‐test. n.s., not significant. (b, c) Cellular stress was evaluated by immunoblot analysis of cleaved caspase‐3 and CHOP after the same FK866 treatment. A known cellular stressor thapsigargin treatment was used as a positive control.

**Figure S2:** Effects of NAD^+^ elevation and suppression on primary cilia in retinal pigment epithelial (RPE)‐1 cells. (a, b) Effects of FK866 treatment (100 nM) on primary cilia in RPE‐1 cells (*n* = 9–10 wells per group). (c, d) Effects of treatment with NMN (100 μM), NR (100 μM), or NAMPT (50 nM) on primary cilia in RPE‐1 cells (*n* = 7–12 wells per group). Data are presented as the mean ± SEM. Statistical significance was determined using a two‐sided unpaired Student's *t*‐test for (a, b) and one‐way ANOVA followed by Tukey's post hoc test for (c, d). All experiments were independently repeated three times. n.s., not significant.

**Figure S3:** SIRT1 deacetylation activity assay. (a) N1 cells were transfected with a mock plasmid, a wild‐type (WT) *SIRT1* plasmid, or a catalytically inactive *SIRT1 H363Y* mutant plasmid (*n* = 3 wells per group). (b) N1 cells were treated with NR (100 μM) in the presence or absence of EX527 (100 μM) for 16 h (*n* = 3 wells per group). (c) N1 cells were transfected with either *siControl* or *siSirt1* (100 pmol). Cells were harvested 48 h after transfection, and SIRT1 deacetylase activity was measured (*n* = 3 wells per group). A.U.; Arbitrary unit. Data are presented as the mean ± SEM. Statistical analyses were performed using one‐way ANOVA followed by Tukey's post hoc test for (a, b) and two‐sided unpaired Student's *t*‐test (c). n.s., not significant. All experiments were independently repeated three times.

**Figure S4:** Confirmation of gene knockdown by quantitative RT‐PCR. (a) Validation of *Sirt1* knockdown based on relative *Sirt1* mRNA expression levels shown in Figure 2d (*n* = 6 wells). (b) *Atg5* mRNA expression levels in the experiment shown in Figure 2k (*n* = 6 wells). (c) *Nampt* mRNA expression levels in the experiment shown in Figure 3d (*n* = 6 wells). (d) Validation of *Ift88* knockdown based on relative *Ift88* mRNA expression levels in the experiments shown in Figures 3e,f, 4d–h, and 5a,c,e (*n* = 3 wells). (e) Validation of *Ift20* knockdown based on relative *Ift20* mRNA expression levels in the experiments shown in Figures 3e–f, 4d–h, and 5a,c,e (*n* = 6 wells). (f) Validation of *Chmp4b* knockdown based on relative *Chmp4b* mRNA expression levels in the experiments shown in Figure 5b,c,e. (*n* = 6 wells). Data are presented as the mean ± SEM. Statistical analyses were performed using two‐sided unpaired Student's *t*‐test. All experiments were independently repeated two times.

**Figure S5:** Ciliary changes in cells transfected with siRNAs targeting various genes. N1 cells were transfected with siRNAs targeting *Atg5* (a), *Nampt* (b), *Ift88* (c), *Ift20* (d), or *Chmp4b* (e) and maintained under serum‐starved conditions for 48 h. Ciliogenesis was assessed by quantifying primary cilium length and the percentage of ciliated cells (*n* = 5 wells per group). Scale bar, 10 μm. Statistical analyses were performed using a two‐sided unpaired Student's *t*‐test. n.s., not significant. All experiments were independently repeated three times.

**Figure S6:** Effects of NMN on TRPML1 agonist‐induced lysosomal Ca^2+^ release in *Ift20*‐depleted N1 cells. Lysosomal Ca^2+^ release induced by the TRPML1 agonist ML‐SA1 was assessed by measuring TRPML1‐GCaMP fluorescence intensity in control and *Ift20*‐depleted N1 cells treated with or without NMN (*n* = 15–22 cells per group). Bar graphs show the mean area under the curve (AUC), and error bars indicate the SEM. Statistical analyses were performed using one‐way ANOVA followed by Tukey's post hoc test.

**Figure S7:** Confirmation of molecular interaction between TRPML1 and CHMP4B. N1 cells were cotransfected with mCherry‐CHMP4B and TRPML1‐MYC. Cell lysates were subjected to immunoprecipitation (IP) using an anti‐CHMP4B antibody, followed by immunoblotting (IB) with antibodies against CHMP4B and TRPML1 to assess their physical interaction. All experiments were independently repeated three times.

**Figure S8:** Effect of autophagy inhibition on basal LC3 accumulation induced by *Ift88* knockdown. N1 cells were transfected with *siControl*, *siIft88*, or *siAtg5*, either alone or in combination, for 48 h. Autophagy was assessed by quantifying the number of LC3 puncta (*n* = 5 wells per group). Data are presented as the mean ± SEM. Statistical analyses were performed using one‐way ANOVA followed by Tukey's post hoc test. The experiment was independently repeated twice.

**Figure S9:** Validation of MBH‐targeted AAV‐*shIft88*‐mediated *Ift88* knockdown and primary cilia disruption in aged mice. (a) Validation of AAV‐*shIft88* delivery to the mediobasal hypothalamus (MBH) of aged mice. Representative images show eGFP fluorescence at the bilateral MBH injection sites and primary cilia within eGFP‐positive MBH regions (*n* = 3–4 mice per group). Scale bar, 200 μm. (b) AAV‐*shIft88* treatment markedly reduced primary cilia length but did not significantly alter the percentage of ciliated cells compared with AAV‐*shControl* treatment. (c, d) Quantitative RT‐PCR (c; *n* = 3–4 mice per group) and western blot analyses (d; *n* = 3 mice per group) confirmed reduced *Ift88* mRNA and IFT88 protein levels, respectively, following AAV‐*shIft88* injection. Bar graphs show the mean values, and error bars indicate the SEM. Statistical analyses were performed using one‐way ANOVA followed by Tukey's post hoc test.

**Figure S10:** Measurement of primary cilium length using ZEN software. (a) Raw fluorescence images were opened in ZEN software, and AC3‐positive primary cilia were identified based on the AC3 fluorescence signal. (b, c) For visualization, identical display settings were applied to all images to enhance the visibility and comparability of ciliary signals. (d) Individual primary cilia were manually identified, and a line was drawn along the entire length of each cilium using ZEN software. (e) The calibrated length of each primary cilium was then automatically calculated by the software.

**Table S1:** Primer sequences used for RT‐PCR.

## Data Availability

All data supporting this study's findings are included in the article and its Supporting Information. Further information and requests for resources and reagents should be directed to Min‐Seon Kim. Source data are provided with this paper.
