# Supplementary material for: A Cilia–Centered Nicotinamide Adenine Dinucleotide (NAD +) Signaling Network Regulates Hypothalamic Lysosomal Homeostasis and Metabolic Aging
Source: Aging Cell. 2026 Oct 20;25(11):e70745. doi: 10.1111/acel.70745 (PMC13684318; doi:10.1111/acel.70745)
Supplement: Supplementary file 1 [file ACEL-25-e70745-s001.pdf]

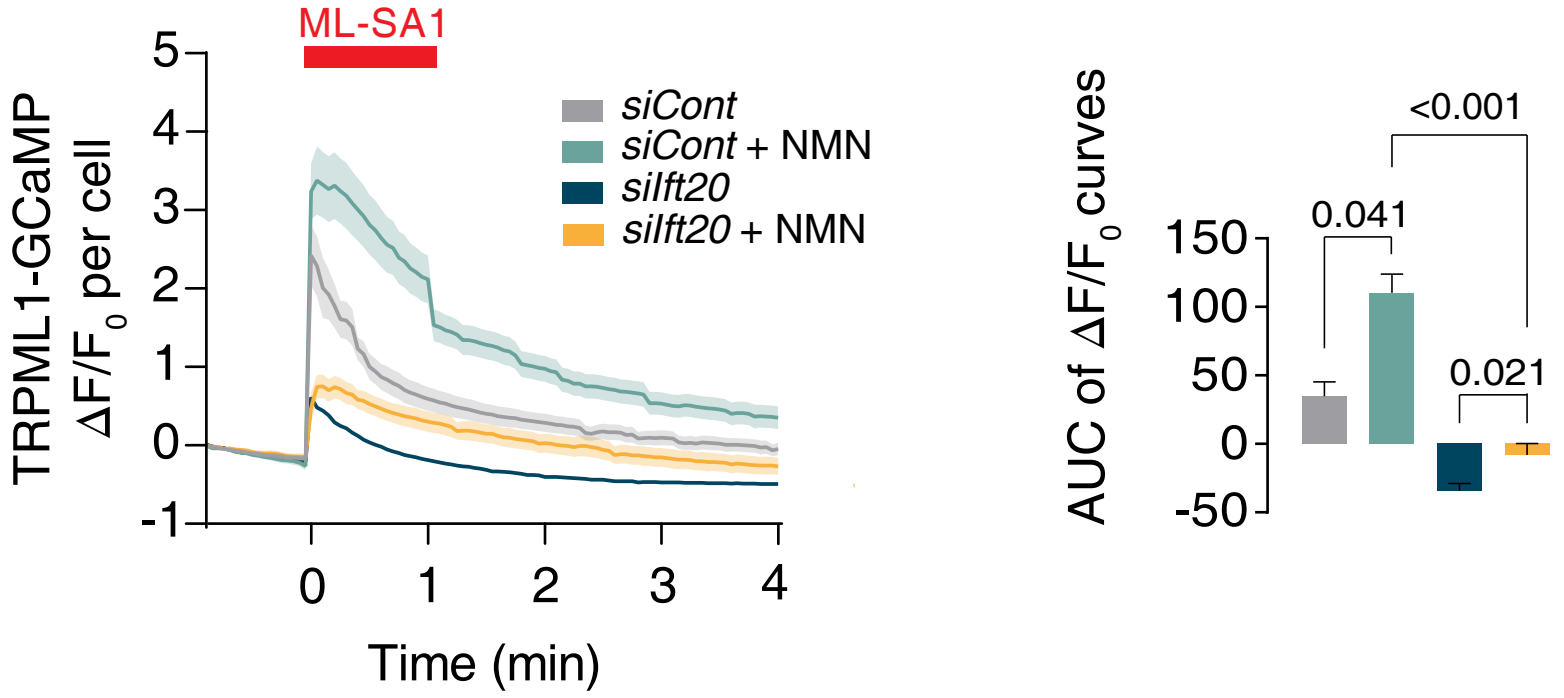

ML-SA1
TRPML1-GCaMP
ΔF/F0 per cell
5
4
3
2
1
0
-1
0
1
2
3
4
Time (min)
siCont
siCont + NMN
siIft20
siIft20 + NMN
AUC of ΔF/F0 curves
150
100
50
0
-50
0.041
<0.001
0.021
