# Supplementary material for: A Cilia–Centered Nicotinamide Adenine Dinucleotide (NAD +) Signaling Network Regulates Hypothalamic Lysosomal Homeostasis and Metabolic Aging
Source: Aging Cell. 2026 Oct 20;25(11):e70745. doi: 10.1111/acel.70745 (PMC13684318; doi:10.1111/acel.70745)
Supplement: Supplementary file 2 [file ACEL-25-e70745-s002.pdf]

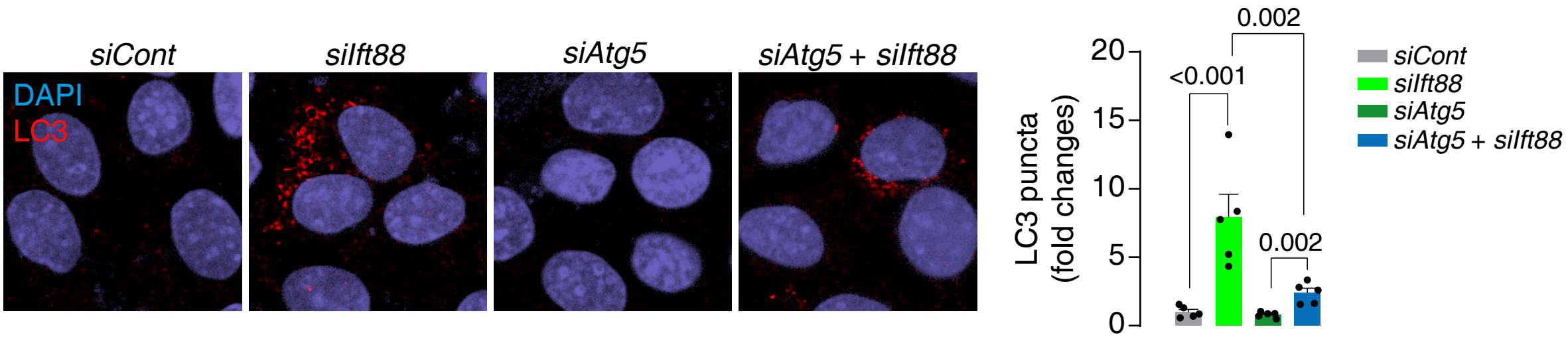
siCont
siIft88
siAtg5
siAtg5 + siIft88
DAPI
LC3
LC3 puncta
(fold changes)
0
5
10
15
20
<0.001
0.002
0.002
siCont
siIft88
siAtg5
siAtg5 + siIft88
