# Supplementary material for: A Cilia–Centered Nicotinamide Adenine Dinucleotide (NAD +) Signaling Network Regulates Hypothalamic Lysosomal Homeostasis and Metabolic Aging
Source: Aging Cell. 2026 Oct 20;25(11):e70745. doi: 10.1111/acel.70745 (PMC13684318; doi:10.1111/acel.70745)
Supplement: Supplementary file 3 [file ACEL-25-e70745-s003.pdf]

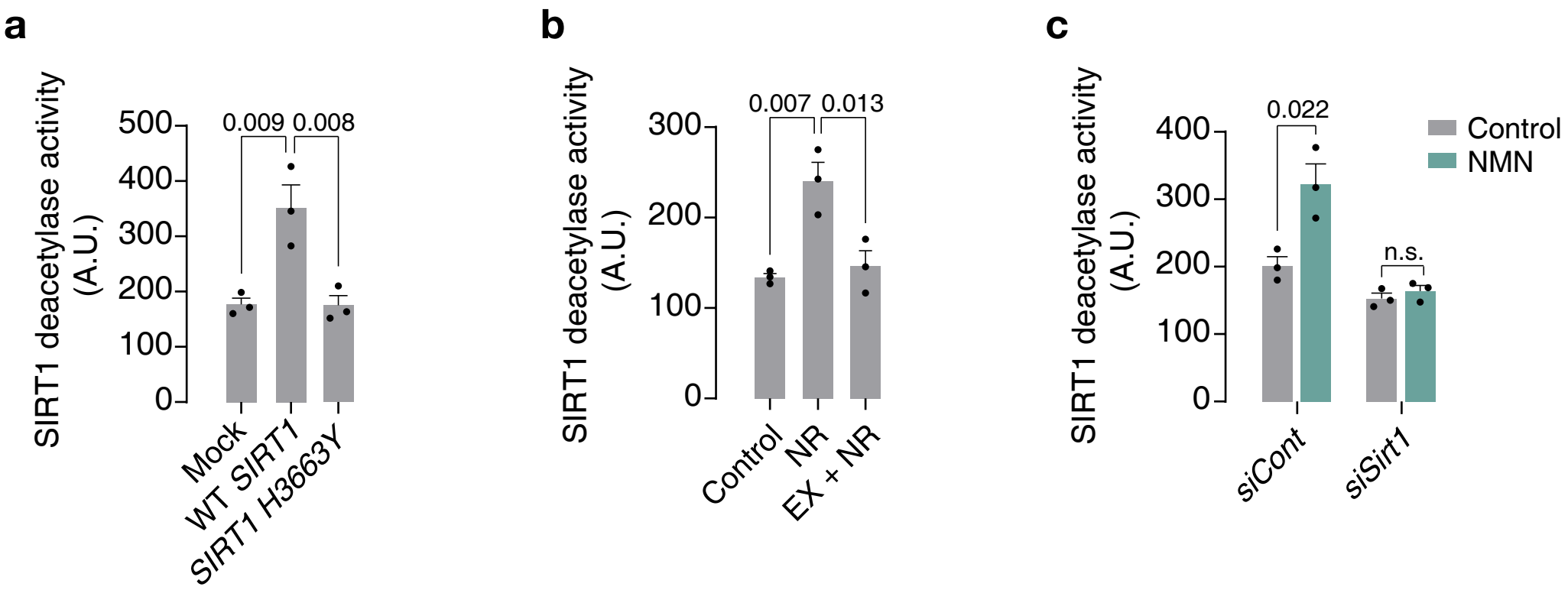
a
SIRT1 deacetylase activity (A.U.)
500
400
300
200
100
0
0.009
0.008
Mock
WT SIRT1
SIRT1 H3663Y
b
SIRT1 deacetylase activity (A.U.)
300
200
100
0
0.007
0.013
Control
NR
EX + NR
c
SIRT1 deacetylase activity (A.U.)
400
300
200
100
0
0.022
n.s.
Control
NMN
siCont
siSirt1
