# Supplementary material for: A Cilia–Centered Nicotinamide Adenine Dinucleotide (NAD +) Signaling Network Regulates Hypothalamic Lysosomal Homeostasis and Metabolic Aging
Source: Aging Cell. 2026 Oct 20;25(11):e70745. doi: 10.1111/acel.70745 (PMC13684318; doi:10.1111/acel.70745)
Supplement: Supplementary file 4 [file ACEL-25-e70745-s004.pdf]

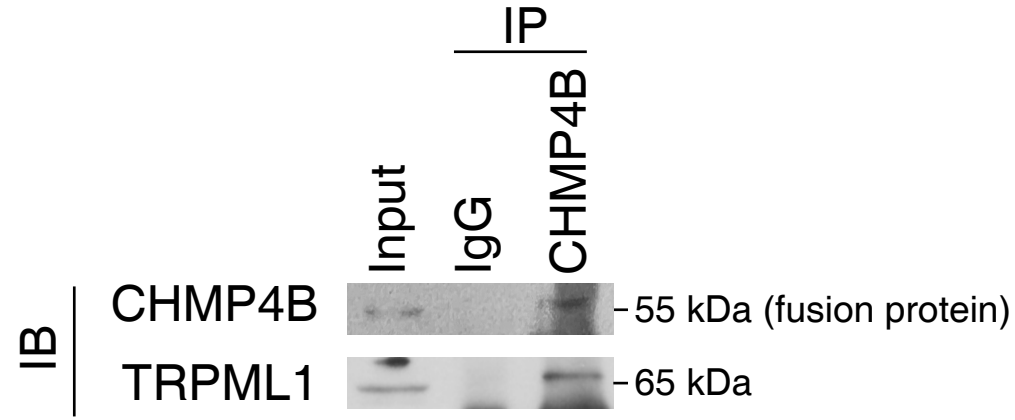
IP
Input
IgG
CHMP4B
IB
CHMP4B
TRPML1
55 kDa (fusion protein)
65 kDa
