# Supplementary material for: A Cilia–Centered Nicotinamide Adenine Dinucleotide (NAD +) Signaling Network Regulates Hypothalamic Lysosomal Homeostasis and Metabolic Aging
Source: Aging Cell. 2026 Oct 20;25(11):e70745. doi: 10.1111/acel.70745 (PMC13684318; doi:10.1111/acel.70745)
Supplement: Supplementary file 5 [file ACEL-25-e70745-s005.pdf]

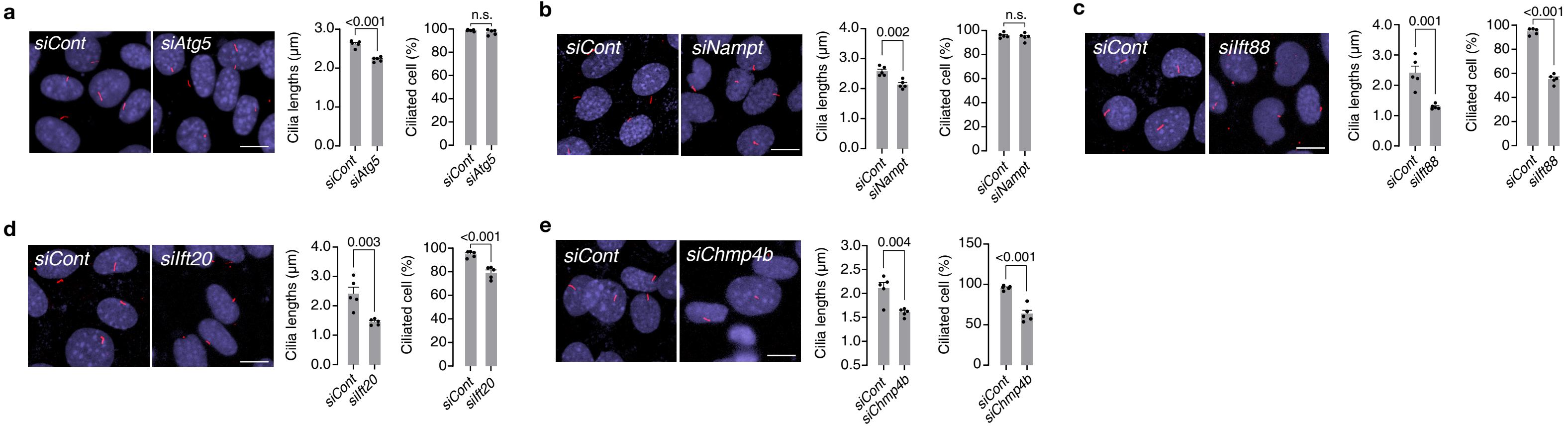

a
siCont
siAtg5
Cilia lengths (μm)
0.0
1.0
2.0
3.0
<0.001
siCont
siAtg5
Ciliated cell (%)
0
20
40
60
80
100
n.s.
siCont
siAtg5
b
siCont
siNampt
Cilia lengths (μm)
0.0
1.0
2.0
3.0
4.0
0.002
siCont
siNampt
Ciliated cell (%)
0
20
40
60
80
100
n.s.
siCont
siNampt
c
siCont
siIft88
Cilia lengths (μm)
0.0
1.0
2.0
3.0
4.0
0.001
siCont
siIft88
Ciliated cell (%)
0
20
40
60
80
100
<0.001
siCont
siIft88
d
siCont
siIft20
Cilia lengths (μm)
0.0
1.0
2.0
3.0
4.0
0.003
siCont
siIft20
Ciliated cell (%)
0
20
40
60
80
100
<0.001
siCont
siIft20
e
siCont
siChmp4b
Cilia lengths (μm)
0.5
1.0
1.5
2.0
2.5
3.0
0.004
siCont
siChmp4b
Ciliated cell (%)
0
50
100
150
<0.001
siCont
siChmp4b
