# Supplementary material for: A Cilia–Centered Nicotinamide Adenine Dinucleotide (NAD +) Signaling Network Regulates Hypothalamic Lysosomal Homeostasis and Metabolic Aging
Source: Aging Cell. 2026 Oct 20;25(11):e70745. doi: 10.1111/acel.70745 (PMC13684318; doi:10.1111/acel.70745)
Supplement: Supplementary file 6 [file ACEL-25-e70745-s006.pdf]

a

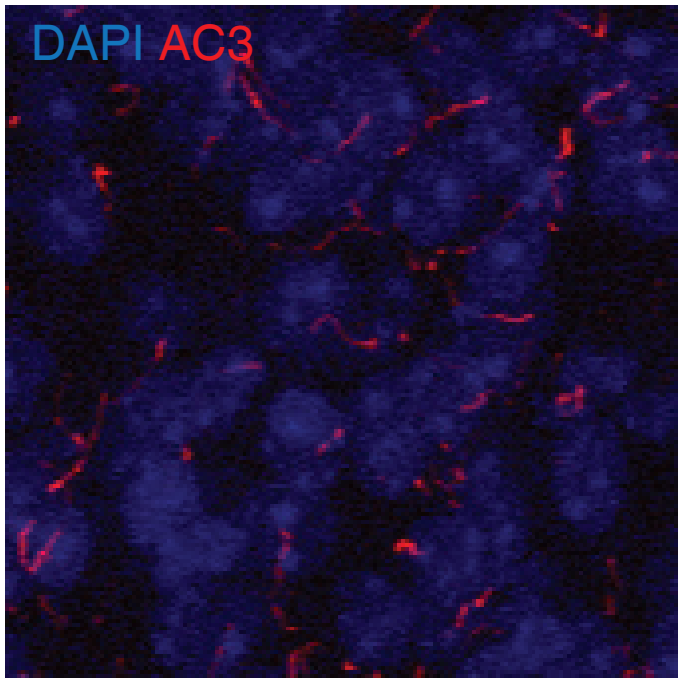

Raw image

b

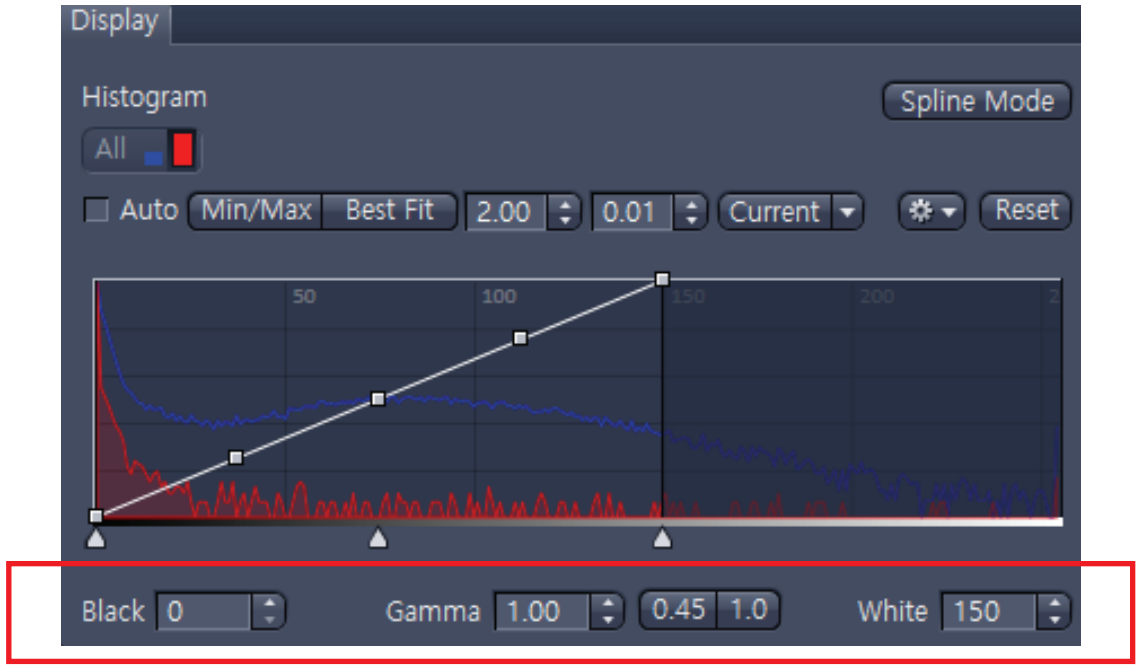

Consolidated view of the ZEN display setting panel

c

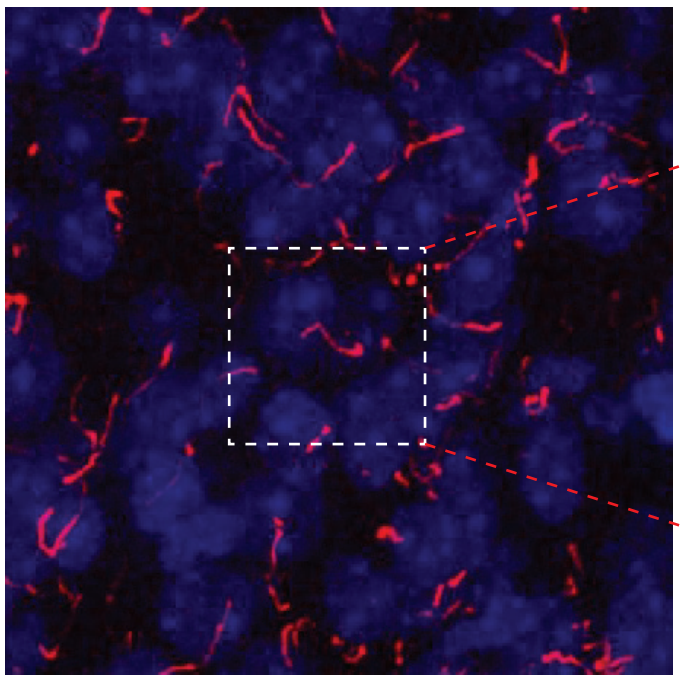

Adjusted image

d

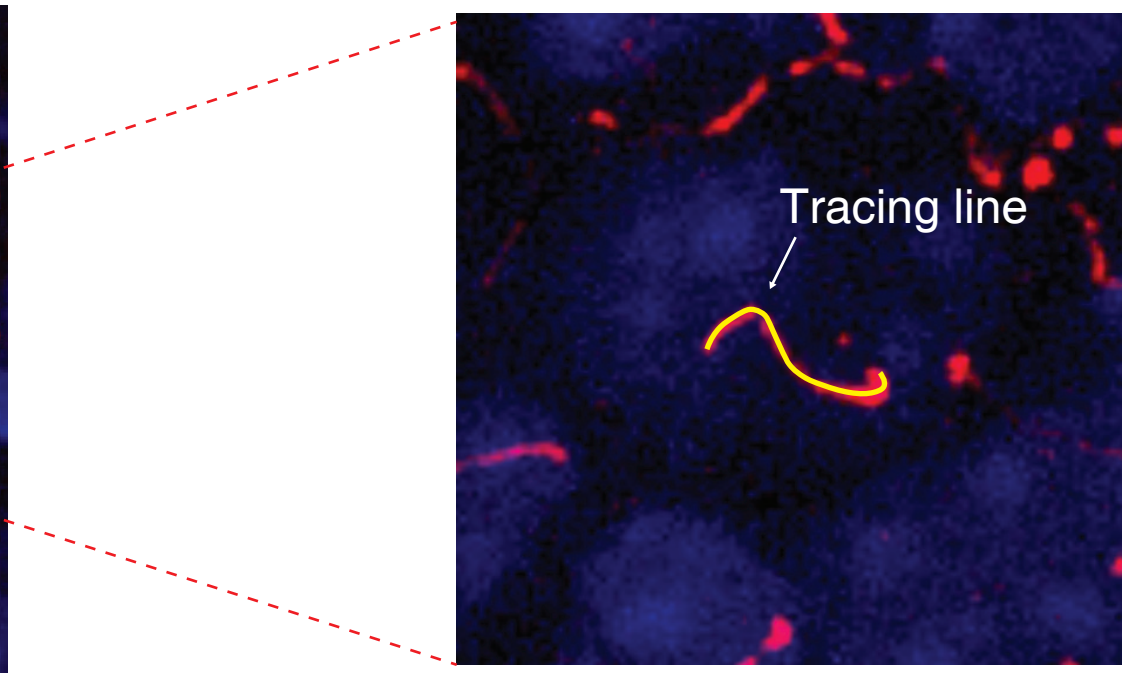

End-to-end length measurement of an AC3-positive cilium using ZEN.

e

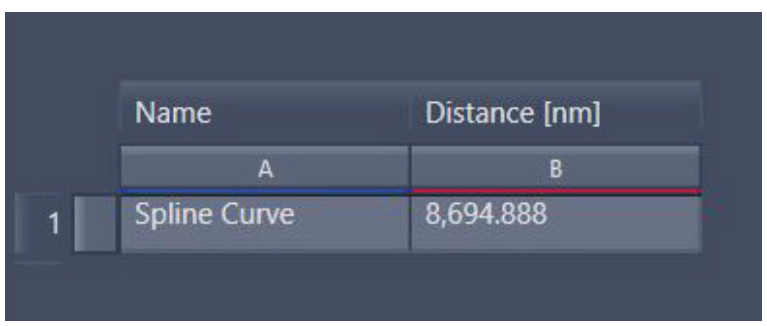

	Name	Distance [nm]
	A	B
1	Spline Curve	8,694.888

ZEN output table showing the automatically calculated calibrated ciliary length.
