# Supplementary material for: A Cilia–Centered Nicotinamide Adenine Dinucleotide (NAD +) Signaling Network Regulates Hypothalamic Lysosomal Homeostasis and Metabolic Aging
Source: Aging Cell. 2026 Oct 20;25(11):e70745. doi: 10.1111/acel.70745 (PMC13684318; doi:10.1111/acel.70745)
Supplement: Supplementary file 7 [file ACEL-25-e70745-s007.pdf]

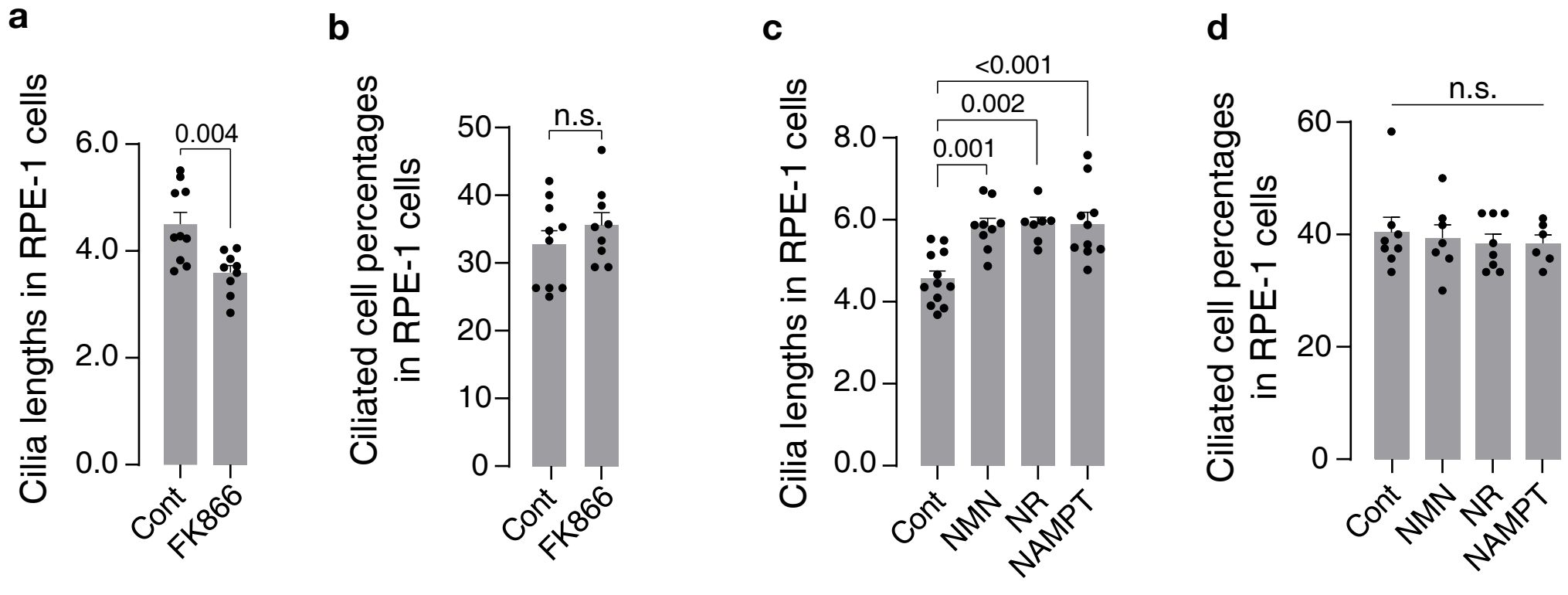

a
Cilia lengths in RPE-1 cells
6.0
4.0
2.0
0.0
0.004
Cont
FK866
b
Ciliated cell percentages in RPE-1 cells
50
40
30
20
10
0
n.s.
Cont
FK866
c
Cilia lengths in RPE-1 cells
8.0
6.0
4.0
2.0
0.0
<0.001
0.002
0.001
Cont
NMN
NR
NAMPT
d
Ciliated cell percentages in RPE-1 cells
60
40
20
0
n.s.
Cont
NMN
NR
NAMPT
