# Supplementary material for: A Cilia–Centered Nicotinamide Adenine Dinucleotide (NAD +) Signaling Network Regulates Hypothalamic Lysosomal Homeostasis and Metabolic Aging
Source: Aging Cell. 2026 Oct 20;25(11):e70745. doi: 10.1111/acel.70745 (PMC13684318; doi:10.1111/acel.70745)
Supplement: Supplementary file 8 [file ACEL-25-e70745-s008.pdf]

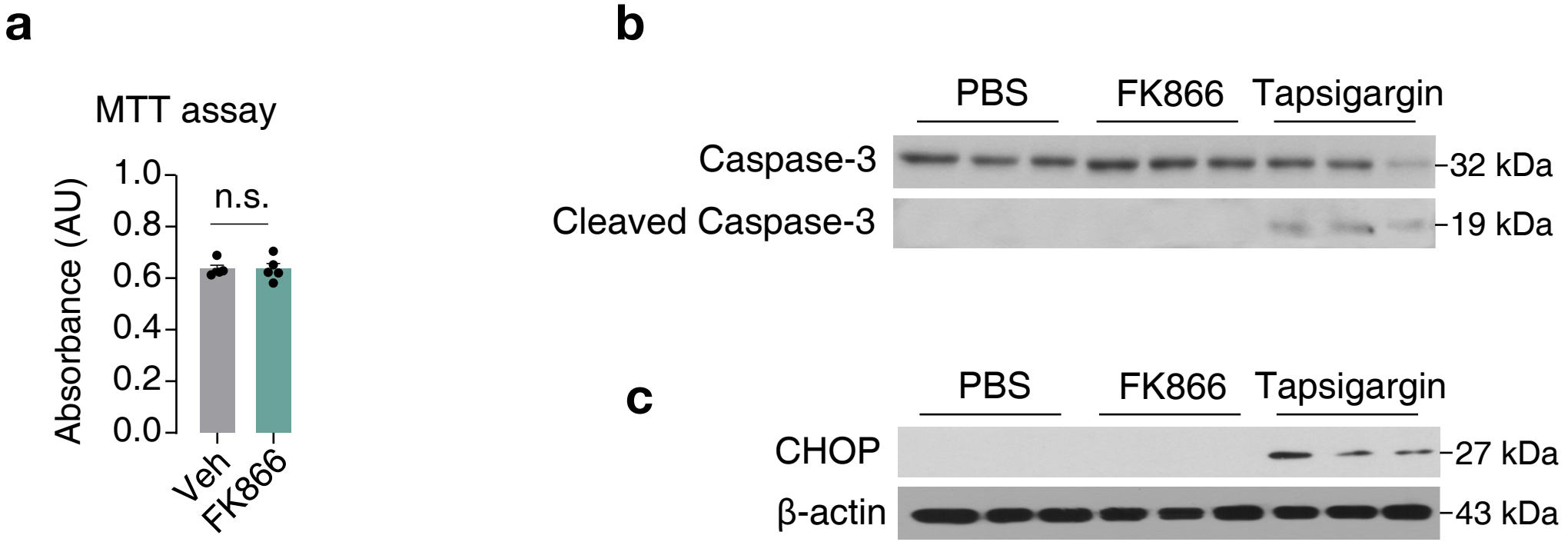
a
MTT assay
Absorbance (AU)
1.0
0.8
0.6
0.4
0.2
0.0
n.s.
Veh
FK866
b
PBS
FK866
Tapsigargin
Caspase-3
Cleaved Caspase-3
32 kDa
19 kDa
c
PBS
FK866
Tapsigargin
CHOP
β-actin
27 kDa
43 kDa
