# Supplementary material for: A Cilia–Centered Nicotinamide Adenine Dinucleotide (NAD +) Signaling Network Regulates Hypothalamic Lysosomal Homeostasis and Metabolic Aging
Source: Aging Cell. 2026 Oct 20;25(11):e70745. doi: 10.1111/acel.70745 (PMC13684318; doi:10.1111/acel.70745)
Supplement: Supplementary file 9 [file ACEL-25-e70745-s009.pdf]

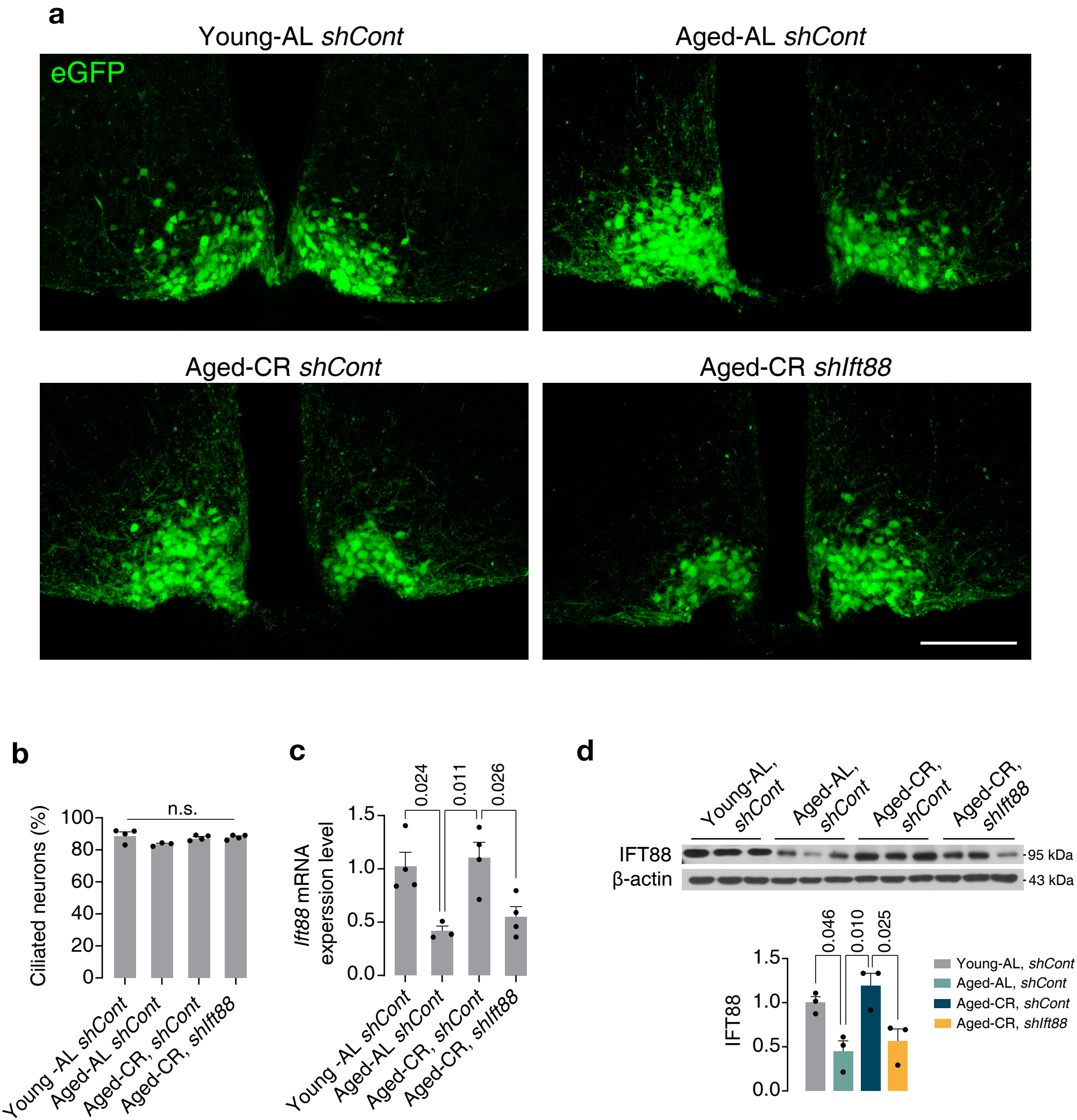

a
Young-AL shCont
Aged-AL shCont
eGFP
Aged-CR shCont
Aged-CR shIft88
b
Ciliated neurons (%)
n.s.
100
80
60
40
20
0
Young -AL shCont
Aged-AL shCont
Aged-CR, shCont
Aged-CR, shIft88
c
Ift88 mRNA expression level
0.024
0.011
0.026
1.5
1.0
0.5
0.0
Young -AL shCont
Aged-AL shCont
Aged-CR, shCont
Aged-CR, shIft88
d
Young-AL, shCont
Aged-AL, shCont
Aged-CR, shCont
Aged-CR, shIft88
IFT88
β-actin
95 kDa
43 kDa
IFT88
0.046
0.010
0.025
1.5
1.0
0.5
0.0
Young-AL, shCont
Aged-AL, shCont
Aged-CR, shCont
Aged-CR, shIft88
