# Supplementary material for: A Cilia–Centered Nicotinamide Adenine Dinucleotide (NAD +) Signaling Network Regulates Hypothalamic Lysosomal Homeostasis and Metabolic Aging
Source: Aging Cell. 2026 Oct 20;25(11):e70745. doi: 10.1111/acel.70745 (PMC13684318; doi:10.1111/acel.70745)
Supplement: Supplementary file 10 [file ACEL-25-e70745-s010.pdf]

Table S1. Primer sequences used for RT-PCR.

Gene	Forward (5'→ 3')	Reverse (3'→ 5')
Ift20	AGGCAGGGCTGCATTTTGAT	CAAGCTCAATTAGACCACCAACA
Ift27	CTGGTGCAGATGTTCCGCA	GTGTCAAGAACTGGCACTGTC
Ift54	GAGGGTGAAGGACCGAGAC	GTCGAGGACCTAGATGCTCC
Ift88	TGAGGACGACCTTTACTCTGG	CTGCCATGACTGGTTCTCACT
Ift144	CAGTATTCACGGGCACTAAAGC	GTACTTGGCGTCCTTTGGC
Kif3a	ATGCCGATCAATAAGTCGGAGA	GTTCCCCTCATTTCATCCACG
Kif3b	CGGCCCATGAATGGCAAAG	CCAATCATACACAGCGTCGAAG
Rfx1	GGCAGCCAGAAGCAGTATGT	GCACCTTCCGATACGGTGA
Rfx2	AGACCCTCAGCTTTACGCC	GTGCCACCTGGAGTCTCAAA
Rfx3	CACGGAATCAAGCAATCTGAGT	GGGGATGAGAATCTCCACCAAA
Rfx4	CACAGAGAGCTGGATTGAAAGAT	CTGAGTGTCGTTCTTCTCACAG
Rfx7	TGCTTTAGGAAGTAGCCGACA	CCATACTGGAGCTTATTGGAGTG
Nampt	GCAGAAGCCGAGTTCAACATC	TTTTCACGGCATTCAAAGTAGGA
Chmp4b	ATGTCGGTGTTCGGGAAGC	CGGCGCGTTTATTTTTGGTG
Atg5	TGTGCTTCGAGATGTGTGGTT	GTCAAATAGCTGACTCTTGGCAA
β-actin	GGCTGTATTCCCCTCCATCG	CCAGTTGGTAACAATGCCATGT
Gapdh	AGGTCGGTGTGAACGGATTTG	TGTAGACCATGTAGTTGAGGTCA
