# Supplementary material for: A Cilia–Centered Nicotinamide Adenine Dinucleotide (NAD +) Signaling Network Regulates Hypothalamic Lysosomal Homeostasis and Metabolic Aging
Source: Aging Cell. 2026 Oct 20;25(11):e70745. doi: 10.1111/acel.70745 (PMC13684318; doi:10.1111/acel.70745)
Supplement: Supplementary file 11 [file ACEL-25-e70745-s011.pdf]

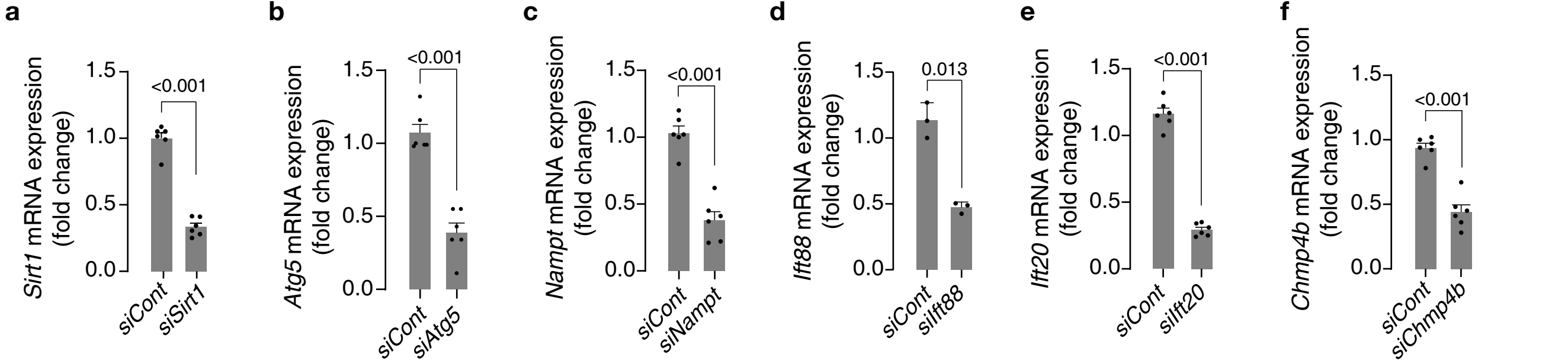
a
b
c
d
e
f
Sirt1 mRNA expression (fold change)
Atg5 mRNA expression (fold change)
Nampt mRNA expression (fold change)
Ift88 mRNA expression (fold change)
Ift20 mRNA expression (fold change)
Chmp4b mRNA expression (fold change)
1.5
1.0
0.5
0.0
<0.001
0.013
siCont
siSirt1
siAtg5
siNampt
siIft88
siIft20
siChmp4b
